# Reduced rotational flows enable the translation of surface-rolling microrobots in confined spaces

**DOI:** 10.1038/s41467-022-34023-z

**Published:** 2022-10-21

**Authors:** Ugur Bozuyuk, Amirreza Aghakhani, Yunus Alapan, Muhammad Yunusa, Paul Wrede, Metin Sitti

**Affiliations:** 1 Physical Intelligence Department, Max Planck Institute for Intelligent Systems, 70569 Stuttgart, Germany; 2 Institute for Biomedical Engineering, ETH Zurich, 8092 Zurich, Switzerland; 3 School of Medicine and School of Engineering, Koç University, Istanbul, 34450 Turkey

**Keywords:** Applied physics, Biomedical engineering, Fluid dynamics

## Abstract

Biological microorganisms overcome the Brownian motion at low Reynolds numbers by utilizing symmetry-breaking mechanisms. Inspired by them, various microrobot locomotion methods have been developed at the microscale by breaking the hydrodynamic symmetry. Although the boundary effects have been extensively studied for microswimmers and employed for surface-rolling microrobots, the behavior of microrobots in the proximity of multiple wall-based “confinement” is yet to be elucidated. Here, we study the confinement effect on the motion of surface-rolling microrobots. Our experiments demonstrate that the locomotion efficiency of spherical microrollers drastically decreases in confined spaces due to out-of-plane rotational flows generated during locomotion. Hence, a slender microroller design, generating smaller rotational flows, is shown to outperform spherical microrollers in confined spaces. Our results elucidate the underlying physics of surface rolling-based locomotion in confined spaces and present a design strategy with optimal flow generation for efficient propulsion in such areas, including blood vessels and microchannels.

## Abstract (web summary)

The effect of geometrical confinement on the locomotion of microrobots is crucial to operating them in real-world applications. Bozuyuk et al. show that the locomotion efficiency of microrollers decreases in confined spaces at high rotation frequencies and propose a slender geometry to overcome this problem.

## Introduction

Locomotion at the microscale requires unique propulsion strategies since viscous forces dominate over inertial forces, where the Reynolds number is much less than unity (Re ≪ 1)^1^. At the low Reynolds number regime, the propulsion mechanisms rely on breaking the flow-field symmetry around the body, which results in a net translational motion^2^. All motile microorganisms, including bacteria, spermatozoa, and algae, break the flow-field symmetry around their bodies by rotating or bending their appendages, such as flagella and cilia, in a non-reciprocal manner^3^. Inspired by such microorganisms, the same fluidic principles have also been applied to achieve synthetic microrobotic propulsion, where the flow-field symmetry around the microfabricated robot bodies has been broken due to their inherent chirality (e.g., helical microswimmers^4,5^). On the other hand, flow-field symmetry around symmetrical (achiral) microrobots can be broken with the help of nearby boundaries^6–8^ when actuated under external fields (e.g., magnetic^6^, acoustic^9^, etc.). Surface-rolling microrobots, i.e., surface microrollers, are a practical example of symmetry-breaking surface propulsion, where the flow-field symmetry is broken with the rotation of the particle body by the presence of a near wall^10,11^. As a result of the magnetically induced rotation, the particle body experiences different hydrodynamic mobilities at the bottom and the top region, resulting in translational motion^12,13^. Surface-enabled rolling locomotion has been shown to produce strong propulsion, generate far greater translational velocities than chiral microrobots and overcome high fluidic flows, including the blood flow.^10,14–17^

While microrobots are almost exclusively envisioned to work in confined microenvironments (e.g., small blood vessels and porous tissue matrices), the effect of geometrical confinement on their propulsion is yet to be elucidated. Essentially, the degree of confinement from the surrounding boundaries is a decisive factor for the locomotion of microrobots due to the increased hydrodynamic interactions with the outer boundaries^18–21^. In a previous study, a decreased propulsion efficiency of surface microrollers in microchannels with decreasing height was noticed^14^. Therefore, a systematic study of microrobotic propulsion in confined environments is required to elucidate the underlying principles of confinement effects on propulsion at the microscale. Apart from the confinement height, several other factors also come into play for the locomotion of surface microrollers in confined spaces, such as rotation frequency (i.e., out-of-plane rotation of the body to achieve translational motion), the shape of the rotating body (e.g., spherical body or rod-like body), and confinement directions (e.g., vertical vs lateral confinement). Exploring the effects of these factors from a fluid dynamics perspective would provide fundamental knowledge of attaining optimal propulsion schemes for next-generation microrobot designs.

**Fig. 1:**
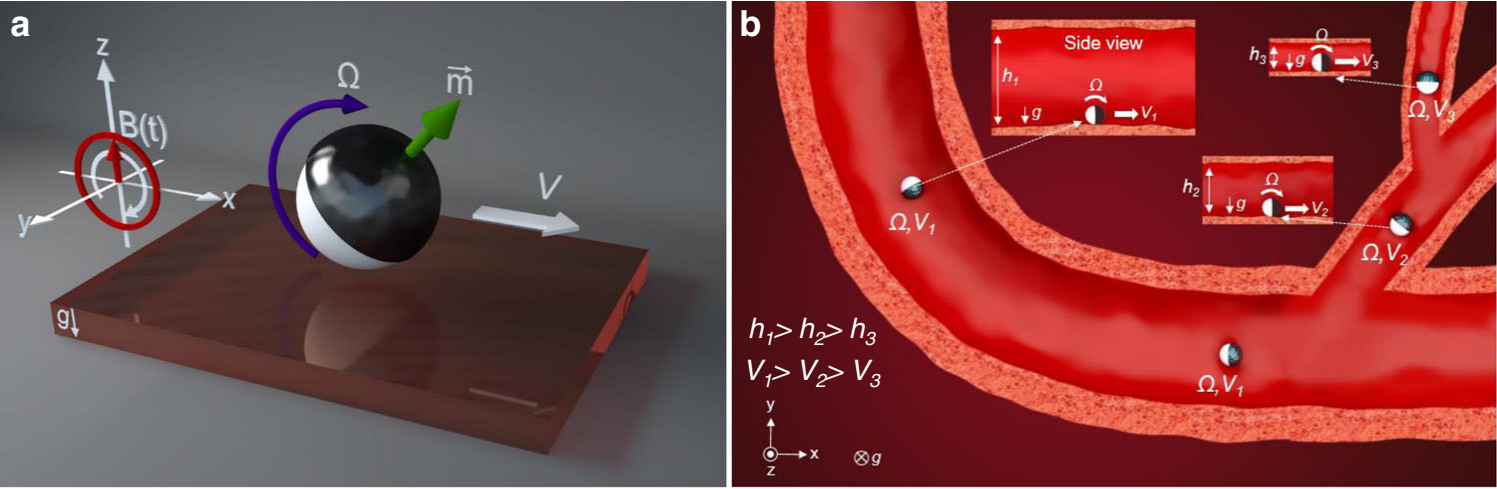
Symmetry-breaking, rolling propulsion of spherical magnetic microrobots in confined spaces. **a** The surface microrollers are composed of 10.8 μm-sized magnetic spherical Janus microparticles actuated with rotating external magnetic fields; the particle rotation results in translational motion by breaking flow-field symmetry by the presence of a near wall. **b** Schematic depicting the microrollers moving in cylindrical microchannels. The microroller translational speed declines as the confinement increases (smaller *h*) due to the out-of-plane rotational flows created.

Here, we systematically investigate the effect of confinement on the motion of the surface microrollers (Fig. 1a) and show rotational flows generated by the microrollers severely impede their locomotion in confined spaces (Fig. 1b). In rectangular microchannels with confinement in the vertical direction, the translational velocity of spherical microrollers drastically decreased only at high magnetic rotational actuation frequencies (*f* ~ 180 Hz), whereas the velocity decrease was negligible at low actuation frequencies (*f* = 20 Hz) with the decreasing channel height. We elucidated the underlying mechanisms for the confinement-induced decrease in translational velocity of the microrollers using computational fluidic dynamic (CFD) analyses, revealing the out-of-plane (according to the nearby wall) rotational flows generated by microrollers were the main contributing factor, rather than the generated translational flows. We also demonstrated that the confinement effect becomes even more severe in circular microchannels due to the additional confinement in the lateral direction. Furthermore, we observed that the microrollers could no longer locomote in the intended actuation direction in circular microchannels; instead, they reversed the locomotion direction at high rotation frequencies. We further tested slender/anisotropic microrollers, generating much smaller rotational flows, and showed their unimpeded and efficient locomotion in the same microchannels to validate our findings. Overall, our results provide an improved understanding of micron-scale locomotion in confined spaces; therefore, the fluidic principles revealed here can be used to revise the conventional microrobotic propulsion mechanisms towards real-world applications of mobile microrobots navigating inside confined fluidic spaces.

## Results

### Microrollers under vertical confinements

**Fig. 2:**
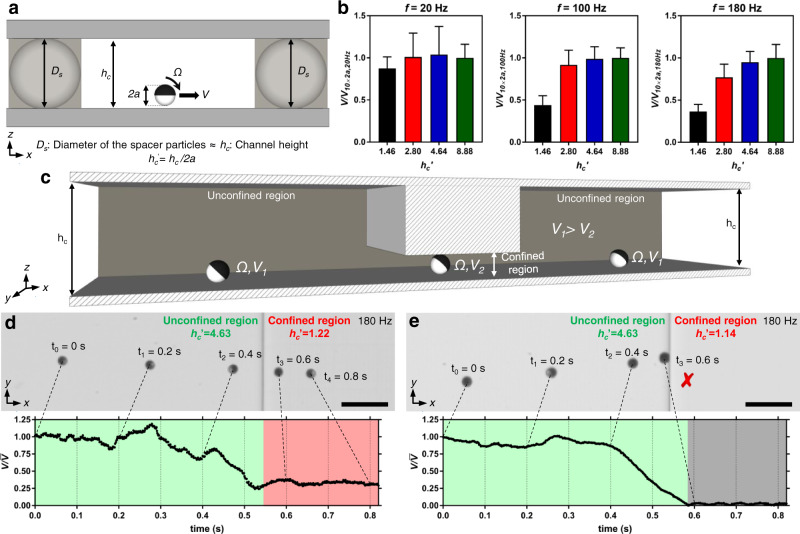
The speed of microrollers dramatically decreases in confined channel experiments. **a** The microrollers were actuated in vertically confined microchannels composed of spacer particles, where the spacer particles’ diameter *(D*_*s*_) defined the channel height (*h*_*c*_), sandwiched between two cover glasses. **b** Effect of the vertical confinement for four different configurations (*h*_*c*_′ = 1.46, 2.80, 4.64, and 8.88) at *f* = 20, 100 and 180 Hz actuation frequencies at 10 mT field amplitude. The translational velocities (*V*) were normalized to the highest translational velocity of individual frequency groups, the least confined case (*h*_*c*_′ = 8.88). The confinement effect was barely visible for the lowest actuation, 20 Hz, while the effect was severe at 180 Hz. The error bars show the standard deviation of the mean. **c** Schematic of the microchannel for the generation of step-like local confinement. Rectangular blocks with different sizes were microfabricated in 3D using two-photon lithography; the channel height subtracted from block height defined the height of the confined region. **d** The velocity of the microroller dramatically reduced upon entry into the confined region (*h*_*c*_′ = *h*_*c*_ */2a* = 1.22) at *f* = 180 Hz. **e** When the confinement was more severe (*h*_*c*_′ = 1.14), the microroller’s velocity dropped to zero and could not even enter the confined region at *f* = 180 Hz. The instantaneous velocity of the microrollers was normalized to the average velocity of the microrollers in less confined region *h*_*c*_′ = 4.63. All scale bars are 50 μm.

The microrollers were composed of 10 μm-sized silica microparticles, half-sputtered with a 1000 nm Ni and 50 nm Au layer, resulting in 10.8 μm average microroller diameter (Fig. 1a and Supplementary Fig. 1). To test the effect of vertical confinement, we prepared rectangular cross-sectioned microchannels, composed of two microscope slides with commercially available microbeads sandwiched in-between as spacers, where the channel height (*h*_*c*_) is defined by the bead diameter (*D*_*s*_) (Fig. 2a). Heights of the microchannels assembled with different beads matched the bead diameters (17, 25, 50, and 100 µm) with slight variations (Supplementary Fig. 2), which could be ascribed to variations within the microparticle sizes. Overall, the propulsion of the microrollers was tested in confined microchannels with varying channel height to microroller diameter (*2a*) ratios, *h*_*c*_′ = *h*_*c*_ */2a* = 1.46, 2.80, 4.64, and 8.88. The microchannels were sufficiently large in the lateral dimensions (*x-y*), in the order of cm-scale. The microrollers were actuated at three different rotation frequencies in phosphate-buffered saline (PBS, 1×.), *f* = *Ω/2π* = 20, 100, and 180 Hz, where *f* = 180 Hz was close to the maximum limit of our electromagnetic actuation system (200 Hz at 10 mT). The microrollers did not show any step-out behavior in any of the confined microchannels (Supplementary Fig. 3); namely, their translational velocity always followed an increasing linear trend with increasing rotation frequency at 10 mT field amplitude. To further prove the microrollers were working in a synchronous rotation regime under the confinements, we analyzed the locomotion of microrollers under more severe confinement, at *h*_*c*_′ = 1.22. We have observed that the magnetic Janus caps always synchronously followed the rotating magnetic field at 20, 100, and 180 Hz at even *h*_*c*_′ = 1.22; namely, the rotation frequency of the microrollers was the same the given magnetic rotation frequency (Supplementary Fig. 4). Therefore, the translational velocity changes of the microrollers was due to the hydrodynamic events that occurred in the confined channels, not the step-out effect.

The vertical confinement effect on the microrollers (i.e., a relative decrease in translational velocity) was not evident at low actuation frequencies (*f* = 20 Hz), while it was very prominent at *f* = 180 Hz (Fig. 2b). The relative translational velocity decrease was only ∼10% for *f* = 20 Hz for the most severe confinement case of *h*_*c*_′ = 1.46, whereas it was ∼65% at *f* = 180 Hz (Fig. 2b). Accordingly, the absolute translational velocity changes were greater at higher rotation frequencies (Supplementary Fig. 3). Moreover, while the confinement effect was evident in almost all confinements at 180 Hz, there was a retarded trend with decreasing actuation frequencies (Fig. 2b). The microrollers under confinements were able to break symmetry to a lower extent, and thus had lower translational velocity in the confined environments.

To further demonstrate the vertical confinement effect in real-time, we microfabricated step-like blocks at different heights on the top side of the microchannels, creating local vertical confinements (Fig. 2c). The microrollers were driven from the less confined region (*h*_*c*_′ = 4.63) to the more confined region (*h*_*c*_′ = 1.22 and 1.14) at 180 Hz, and changes in the translational velocities (*V*) were recorded against time (Fig. 2d, e, Supplementary Fig. 5). The translational velocity of the microrollers dramatically decreased, almost fourfold, upon entry into the more confined region (*h*_*c*_′ = 1.22) (Fig. 2d, Supplementary Fig. 5a, b and Supplementary Movie 1). When the confinement was even more severe (*h*_*c*_′ = 1.14), the microrollers could not even enter the confined region, since the translational velocity dropped to zero before reaching the boundary of the confinement entrance, despite continuous rotation of the microrollers (Fig. 2e, Supplementary Fig. 5c, d and Supplementary Movie 1). Overall, the experiments showed that the vertical confinement effect (i) is more evident with an increasing rotation frequency, (ii) is instantly felt by the microrollers with a sudden change in the channel height, (iii) and could also prevent the microrollers entering the confined regions.

### Microrollers under irregular vertical confinements

**Fig. 3:**
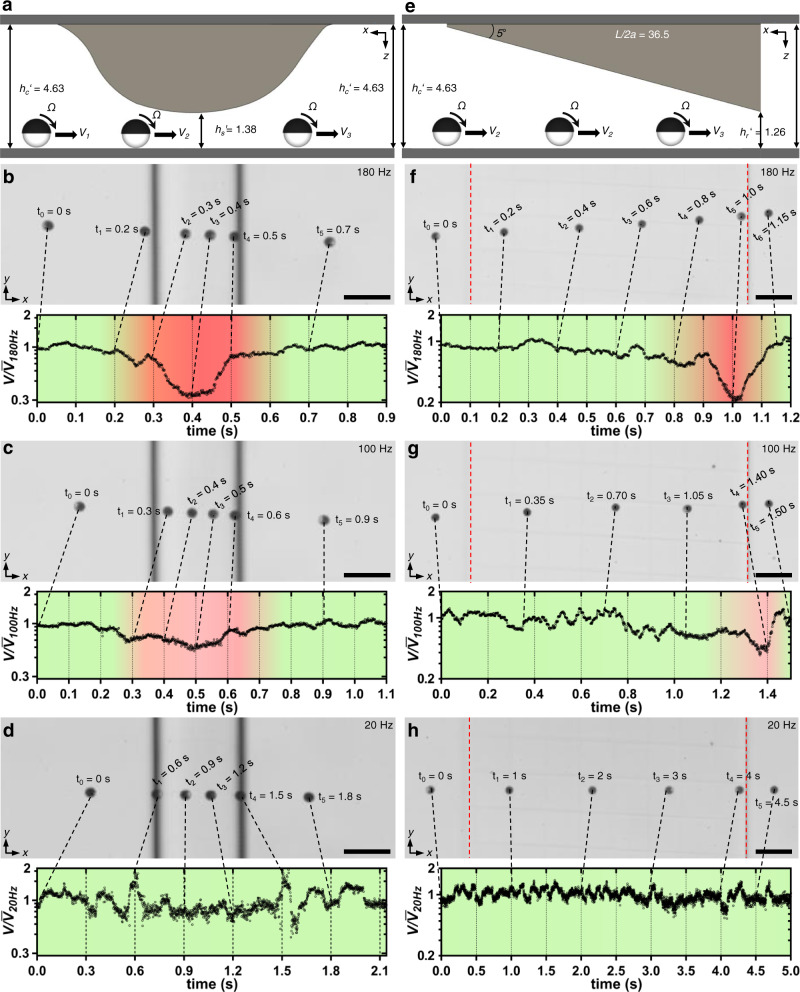
Microroller locomotion experiments under irregular vertical confinements. **a** A half-sphere-like structure was microfabricated using two-photon lithography and placed upside down from the top of the microchannel. The translational velocity of the microroller changed depending on its position under the confinement. **b**–**d** The time-lapse images and time versus transitional velocity graphs of microrollers actuated at different rotational frequencies, *f* = 180, 100, and 20 Hz, under the half-sphere-like confinement. At the highest rotation frequency (*f* = 180 Hz), the translational velocity of the microroller drastically changed depending on the rate of confinement. This indicated that the microroller was very sensitive to confinement effects at high frequencies. The velocity differences were less drastic at *f* = 100 Hz and almost vanished at *f* = 20 Hz. **e** The ramp structures were microprinted using two-photon lithography with a 5° ramp angle and *L’* = *L/2a* = 36.5 and *h*_*r*_*’* = 1.26. **f**–**h** The time-lapse images and time versus velocity graphs of microrollers actuated at different rotational frequencies under the ramp confinement. For the highest actuation frequency (*f* = 180 Hz), the confinement effect was not seen until almost at the end of the ramp, where the velocity decreased suddenly started before 27.5 μm before the end of the ramp. Similar to the half-sphere structure, the effect was slightly visible for *f* = 100 Hz and negligible for *f* = 20 Hz. Dashed red lines in time-lapse images indicate the start and endpoints of the ramp. The instantaneous velocity was normalized to the average velocity for all graphs at the specific condition. All scale bars are 50 μm.

To characterize the confinement effect in detail, we microfabricated irregular structures, including a half-sphere and a ramp, on the top side of the microchannels. The translational velocities of the actuated microrollers under the irregular structures were recorded with time and position (Fig. 3 and Supplementary Fig. 6).

The half-sphere-like structure presented maximum confinement of *h*_*s*_′ = 1.38 (Fig. 3a and Supplementary Fig. 6a, c). When the microrollers were actuated under the semi-sphere structure at different actuation frequencies (*f* = 20, 100, and 180 Hz), the confinement effect was more prominent at higher frequencies (Fig. 3b–d, Supplementary Fig. 7), in agreement with the previous results (Fig. 2b). Specifically, the instantaneous translational velocity of the microrollers at *f* = 180 Hz was very sensitive to the degree of confinement, where there was a close match between the velocity profile of the microroller with the geometric shape of the semi-spherical structure (Fig. 3b, Supplementary Fig. 7a and Supplementary Movie 2). On the other hand, the relative translational velocity decrease was less at 100 Hz and almost not visible at 20 Hz (Fig. 3c, d, Supplementary Fig. 7b, c). Furthermore, despite the similarity of the velocity profile and the geometry of the microstructure, a closer inspection of the microroller position and the velocity profile revealed that the translational velocity change was not fully synchronous to the degree of confinement. For example, at t_2_ = 0.3 s (Fig. 3b), even though the microroller was close to the most confined section, its instantaneous translational velocity was still close to the maximum velocity. The instantaneous velocity decrease started after the microroller passed the centerline of the semi-sphere (*h*_*s*_′ = 1.38). The discrepancy between the velocities measured here and the homogenous confinement could be ascribed to the non-planar geometry of the confined region. Since the confinement changes non-linearly in the axis of the microroller translation direction, at each position underneath the half-sphere top wall, the confinement at the back and front of the microroller would be different, resulting in the velocity mismatch with the continuous confinement, shown Fig. 2e.

For the ramp confinement, we microfabricated a ramp-like structure with a 5° angle with a total distance of *L*′ = *L/2a* = 36.5 and maximum confinement of *h*_*r*_′ = *h*_*r*_ */2a* = 1.26 (Fig. 3e and Supplementary Fig. 6b, d). The microrollers were again driven from the less confined section of the microchannel to the confined section underneath the ramp structure (Fig. 3e). The confinement effect was not prominent throughout the ramp at *f* = 180 Hz, except at the end portion. The translational velocity suddenly dropped at the end of the ramp but then recovered after passing the structure (Fig. 3f, Supplementary Fig. 8a). At *f* = 100 and 20 Hz, the confinement effect was less prominent and negligible (Fig. 3g, h, Supplementary Fig. 8b, c), respectively. Overall, the irregular vertical confinement experiments have demonstrated that (i) the microrollers are sensitive to environmental changes, especially at high rotation frequencies, and (ii) the translational velocity can dynamically change within non-monotonic environments despite a constant rotational velocity.

### Hydrodynamic interactions of the microrollers with planar confinements

**Fig. 4:**
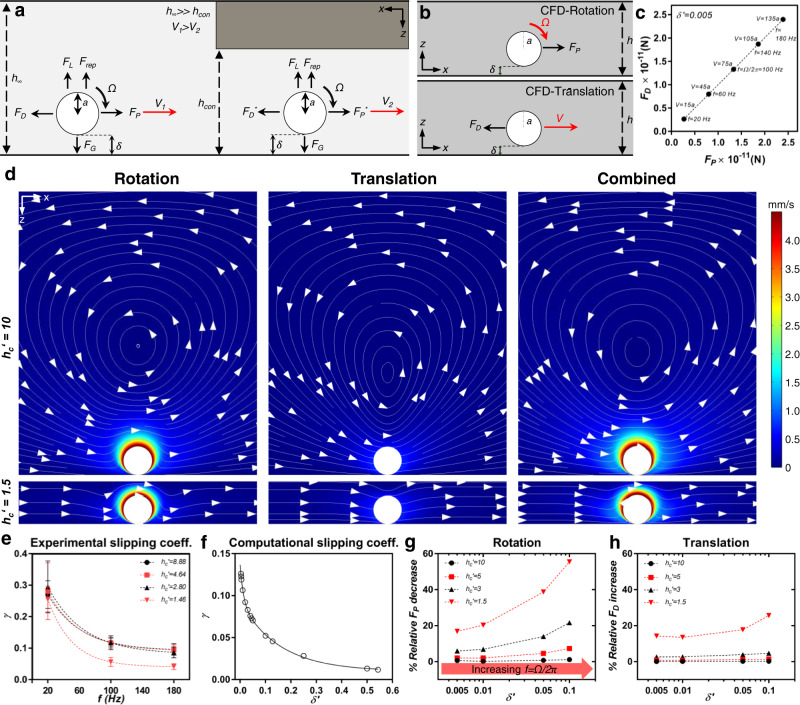
Three-dimensional (3D) computational fluid dynamics (CFD) simulations of the microrollers in vertically confined spaces. **a** Mechanistic explanation of the microroller propulsion without and with vertical confinement. Without confinement, the propulsion force (*F*_*P*_) is balanced out by the drag force (*F*_*D*_) on the *x*-axis, and gravitational forces (*F*_*G*_) are balanced out by repulsion (*F*_*rep*_) and lift forces (*F*_*L*_) on the *y*-axis. *δ* shows the lubrication distance. Under the confinement, the correction factors of Eqs. 1 and 2 change and result in new forces, *F*_P_^*^ and *F*_D_^*^, thus changing the overall microroller translational velocity (*V*). **b** Summary of the CFD simulations. The spherical objects with fixed positions were rotated and translated separately for validation. **c** Validation of the simulations for an extreme lubrication distance condition, *δ*^*’*^ = *δ/a* = *0.005*. The spheres were rotated and then translated with their calculated theoretical speed. The forces acting on the spheres were same but opposite directions (less than 0.5% difference), as they should be in the Stokes regime, demonstrating the validity of the simulations. **d** Flow-fields around the rotating, translating (theoretical speed, corresponding to the rotation frequency) and the combination of two (rotation + translation) at 180 Hz, for different confinements *h*_*c*_′ = 10 and *h*_*c*_′ = 1.5. Colors shows the magnitude of the flow velocity, streamlines and arrows show the direction of the flow. From a qualitative first impression, it was concluded that the rotational flows were more dominant in our system. **e** The experimental slipping coefficients, *γ*, of microrollers under different confinements. The microrollers were more efficient, conversion of rotation to translation, at *f* = 20 Hz, and the efficiencies decreased with increasing rotational frequencies. Error bars show standard deviation of the mean. **f** Universal computational slipping coefficients, *γ*, for different *δ’* ratios at the semi-infinite channel. The slipping coefficients followed a decreased trend with increasing *δ’* as in the experimental slipping coefficients, indicating a close relationship between rotation frequency *(f* = *Ω/2π*) and lubrication distance. The graph is reproduced by Supplementary Fig. 9c. **g** Universal propulsion force decrease graph depending on *δ’* under different confinements of a rotating sphere. The propulsion force decreased with increasing confinement rate, and the increase was very drastic at increasing *δ’* at higher confinement rates. **h** Universal drag force increase graph depending on *δ’* under different confinements of a translating sphere. The drag force increased with increasing confinement rate; however, the increase was not drastic as the rotating sphere case.

The confinement effect on a microroller can be mechanistically explained using the force balances on a microroller in the Stokes regime (rotational and translational *Re* numbers, *Re*_*Ω*_ = 0.02826, *Re*_*V*_ = 0.0063 for *f* = 180 Hz). The rotational motion of a microroller is converted to translational motion due to the presence of a solid near-boundary by breaking the flow-field symmetry around the microroller. The force balance on a microroller without a confinement effect in the Stokes regime is given in Fig. 4a (left microroller). In unconfined or semi-infinite dimensions, on the translational movement axis (*x-*axis, Fig. 4a), the propulsion force (*F*_*P*_) is balanced out by the drag force (*F*_*D*_)^22^:

1 $${F}_{P}=\pi \mu {a}^{2}\varOmega {\xi }_{1}(\delta ^{\prime} )$$

2 $${F}_{D}=6\pi \mu {aV}{\xi }_{2}(\delta ^{\prime} )$$

where *μ* is the dynamic viscosity of the fluid, *a* is the microroller radius, *Ω* is the angular velocity, *V* is the translational velocity, ${\xi }_{1}$ and ${\xi }_{2}$ are wall correcting factors, and ${\delta }^{{\prime} }=\delta /a$ (Fig. 4a) is the dimensionless lubrication distance which experimentally depends on *Ω* and *V*. Since the forces are in balance in the Stokes regime, an expression for the *V* can be derived as:

3 $$V=\,\frac{a\varOmega {\xi }_{1}({\delta }^{{\prime} })}{6{\xi }_{2}({\delta }^{{\prime} })}.$$

Therefore, *V* is the function of *a*, *Ω* and $\delta ^{\prime}$. Increasing *a* and *Ω* would increase the *V*; however, increasing $\delta ^{\prime}$ would decrease *V*. Here, *a* and *Ω* are intrinsic parameters for the microroller system; namely, *a* is the size of the microrollers that is fixed and determined by the fabrication process and *Ω* is an external parameter that can be manipulated with the magnetic actuation system. However, $\delta ^{\prime}$ cannot be externally controlled in our system and deterministically reaches a value depending on the inputs given. The value of $\delta ^{\prime}$ is mainly dependent on the force balance of the *z-*axis (Fig. 4a, left microroller); the gravitational force against buoyancy (*F*_*G*_) is balanced out by the repulsion force (*F*_*rep*_) and the lift force (*F*_*L*_). *F*_*G*_ and *F*_*rep*_ are entirely dependent on the intrinsic parameters of the system, which cannot be externally manipulated; however, the lift force is both functions of *Ω* and *V*, and both are directly proportional to *F*_*L*_^23^.

When a microroller is locomoting in a confined space, the governing forces, *F*_*P*_ and *F*_*D*_, also become dependent on the *h*_*c*_′ (Fig. 4a) due to new hydrodynamic wall effects, which leads to a new force balance with modified correction factors. From the new force balance, *V* can be expressed as follows:

4 $$V=\,\frac{a\varOmega {{\xi }_{1}}^{ * }({\delta }^{{\prime} },\,{{h}_{c}}^{{\prime} })}{6{{\xi }_{2}}^{ * }({\delta }^{{\prime} },\,{{h}_{c}}^{{\prime} })}$$

where ${{\xi }_{1}}^{ * }$ and ${{\xi }_{2}}^{ * }$ are the modified correction factors. Here, the decrease in *h*_*c*_′ would decrease the *V* of the microroller, as evidenced by our experiments. Even though the mechanistic explanation provides insight into the governing parameters of the system and explains the decreased translational velocity, it does not provide any detailed explanation of what type of hydrodynamic interactions cause the force changes. For example, changes in the modified correction factors could be due to both rotational or translational flows generated by the microroller, the elucidation of which requires numerical investigation of the hydrodynamic interactions.

To unravel the nature of the additional forces acting on the microrollers under confinement, we performed three-dimensional (3D) CFD analyses. Since performing a dynamic simulation of the microrollers is computationally expensive, the fixed spheres having *2a* = 10 µm diameter were rotated, translated, or both rotated and translated at different confinement ratios. To validate our finite element simulations, we computed hydrodynamic forces acting on the spheres for two particular cases: rotation and translation (Fig. 4b) at different $\delta ^{\prime}$ according to:

5 $$F=\iint \sigma \cdot n{{{{{\rm{dS}}}}}}$$

where *σ* is the stress tensor, *n* is the outward-pointing normal vector, and S is the particle surface. In the rotating sphere, the total acting force resulted in +*x* direction with clockwise rotation of the body, and this force is essentially *F*_*P*_ (Eq. 1). In the translating sphere, the total acting force resulted in *-x* direction with a translation in *+x* direction, and that force is essentially *F*_*D*_ (Eq. 2). Our simulations have shown quantitative agreement with the results from Goldman et al.^22,24^ with channel dimensions of 40*a* × 40*a* × 40*a* (Supplementary Fig. 9a, b). At these dimensions, the sphere was essentially in a semi-infinite fluid; thus, there was no confinement effect. We performed another set of CFD analyses for further validation, where we separately calculated the forces acting on the rotating and translating spheres at their theoretically matching *Ω* and *V* at $\delta ^{\prime}$ = 0.005. To calculate the theoretical *V* for *Ω*, we used the following equation obtained by curve fitting of $V/a\varOmega$ with respect to $\delta ^{\prime}$ (*R*^2^ = 0.9995, Supplementary Fig. 9c):

6 $$V=a\varOmega \left(0.009708+0.04442180160\,{{\exp }}\left(-78.07\left({\delta }^{{\prime} } \right. \right. \right)+0.08257019840\,{{\exp }}\left(-6.383\left({\delta }^{{\prime} }\right)\right).$$

As mentioned earlier, the acting forces on the microroller must be in balance in the Stokes regime, which has been shown in our CFD analyses, further validating our simulations (Fig. 4c). Of note, the sole aim to derive Eq. 6 was to validate our simulation environment, by comparing the forces generated in rotation and translation simulations for the given input parameters of $\delta ^{\prime}$ and *Ω*. In experiments, $\delta ^{\prime}$, *Ω*, and *V* are also functions of each other, therefore the relation is more complex than Eq. 6. The validation showed that our mesh refinement in the simulations was sufficient to capture the force dynamics, and microrollers with different geometries can also be safely modeled in the simulation environment^24^.

According to the lubrication theory, the combined effect on a simultaneously rotating and translating sphere is the vector summation of two separate effects (only rotating and only translating spheres) due to the linearity of the motion and stress equations^22^. Therefore, we can decompose our system into two separate cases: the rotating sphere and the translating sphere. This way, we can understand which type of flow generated by the microrollers contributes most to the impeded locomotion in confined spaces. To obtain a qualitative understanding, we performed CFD analyses with channel dimensions of 40*a* × 40*a* × 20*a* and 40*a* × 40*a* × 3*a*, which represent slightly and hardly confined configurations. Furthermore, we simulated only rotating, only translating, and rotating and translating spheres at *f* = 180 Hz rotation frequency with its corresponding theoretical *V* for $\delta ^{\prime}$ = 0.005. First, the flow-field magnitudes were much more prominent in the rotating sphere compared to the translating sphere (Fig. 4d). Also, qualitatively, flow-field arrows and magnitudes were very similar between the rotating sphere and the rotating and translating sphere (Rotation and Combined in Fig. 4d). Among these three cases, the translating sphere contributed the least to flow magnitudes around the sphere which implicated the rotational flows could be the main contributor for the decreased *V* in the experiments. However, a quantitative understanding is essential to explain this case thoroughly.

To establish a quantitative understanding, first, we must understand how the rotation frequency, *f* changes $\delta ^{\prime}$, since they are the only contributing variables for *V* (Eq. 3). It has been previously shown that $\delta ^{\prime}$ has increased with increasing *f* for surface microrollers using total internal reflection microscopy^25^. Even though we could not directly measure $\delta ^{\prime}$ in our experimental setup, we deduced that a similar relation between *f* and $\delta ^{\prime}$could also be valid for our microroller system by comparing our experimental and simulation results (Fig. 4e, f). From our experiments, the microroller locomotion was more efficient at lower frequencies, evidenced by the slipping coefficient, $\gamma=V/a\varOmega$ (Fig. 4e), where *γ* = 1 represents pure rolling and *γ* = 0 pure slipping^26,27^. In other words, the microrollers slip less and more efficient at low frequencies, and their efficiency decreased by increasing the rotation frequency (Fig. 4e). This efficiency decrease could only be associated with increasing $\delta ^{\prime}$, as shown previously^25^ and as well as in our simulations (Fig. 4f). Of note, the experimental and computational slipping coefficients were different, which could be due to the no-slip boundary condition applied in the simulations^22^.

In the Stokes regime, there is a linear relationship between rotation frequency, *f*, and the *F*_*P*_ or *V* generated by a microroller at fixed the $\delta ^{\prime}$ values (Eq. 3). However, in our case $\delta ^{\prime}$ increases with increasing *f* and causes non-linearities due to the inverse relationship between *V* and $\delta ^{\prime}$. Therefore, the net positive effect of increasing *f* on *V* is in fact damped by the increase in $\delta ^{\prime}$. To be able to capture the effect of increasing *f* in the simulation environment, ${\delta }^{{\prime} }$ must also be assigned accordingly. In fact, $\delta ^{\prime}$ is the main and only critical parameter in CFD simulations to investigate relative *F*_*P*_ decreases for different confinements, since the relative changes in *F*_*P*_ will be the same at any *f* value at fixed $\delta ^{\prime}$ due to the linearity of the system for *f*. Therefore, ${\delta }^{{\prime} }$ becomes the main parameter analyzing the system for different *f* regions. In CFD simulations, we analyzed the decreases of *F*_*P*_ relative to the semi-infinite case (40*a* × 40*a* × 40*a*) at different confinements (40*a* × 40*a* × *h*_*c*_, *h*_*c*_′ = 1.5, 3, 5 and 10) for $\delta ^{\prime}$*=* 0.005, 0.01, 0.05, and 0.1, where smaller and higher $\delta ^{\prime}$ represent lower and higher *f* regions, respectively. From Fig. 4g, the relative *F*_*P*_ decreases were very drastic for the higher *f* region for *h*_*c*_′ = 1.5, while less drastic for the lower *f* region, in agreement with the experimental results (Fig. 2b). Then, we performed CFD analyses of the translating sphere in the same configurations with a constant *V*, and calculated the relative *F*_*D*_ increase on the sphere. The linear relation still holds for the translating sphere at fixed $\delta ^{\prime}$ in the Stokes regime; the relative changes in *F*_*D*_ still will be the same for different *V*. For a constant *V*, at the high *f* regime (i.e., higher $\delta ^{\prime}$), the relative *F*_*D*_ increase on the sphere was not as drastic as in the case of the rotating sphere (Fig. 4g, h). In addition, it should be noted that *V* is an output in our system that decreases with increased confinement degree (Fig. 2b). Therefore, the confinement effect in Fig. 4h was exaggerated since the *V* was kept at the same value in the simulations. To carefully investigate the translating sphere case, we re-calculated the *V* values (Supplementary Fig. 10a) for each specific case found in the (Fig. 4h) depending on the relative *F*_*P*_ decrease (Fig. 4g). We again found out that the contribution of translational flows was again not as drastic as the contribution of rotational flows (Supplementary Fig. 10b, c). These results indicate that the rotational flows are the mere contributor to the impeded motion of the microrollers under the confinements.

Of note, lift force was negligible in CFD simulations when compared to the *F*_*P*_ or *F*_*D*_ with and without confinements (Supplementary Fig. 11)^27,28^. However, it was previously shown that the lubrication gap increases with increasing rotation frequency for surface microrollers with similar size scales found here^25^. There could still be an experimental lift force pushing the particles away from the surface, which may not be apparent in the simulations due to the no-slip boundary condition^29^. A recent study by Rinehart et al. has demonstrated that a non-trivial lift force emerges from a rotating Janus particle in the Stokes regime due to slip inhomogeneity of the particle body^30^. The increase in lubrication distance could also be attributed to increasing wet friction that occurs at higher translational velocities and rotation frequencies^25–27^. Nevertheless, previous experimental validations^25^ and our experiments indicate that the lubrication distance increases with increased rotation frequency.

Other than continuous confinements analyzed so far, we also assessed the effect of the step-like confinement (Fig. 2c) in the CFD environment. We hypothesize that the hydrodynamic flow field generated by the microroller prevents it to enter the confinement due to increased boundary interactions; the microroller was simply pushed by the wall to the opposite direction of its translation, despite its constant rotation^14^. In other words, the propulsion force generated by a microroller should have decreased in Fig. 2d, e, the latter to a higher extent. To elucidate the effect of step-like confinement in this study, we performed additional CFD analyses. We mimicked the experimental environment, where we changed the position of the microroller with respect to the step-like confinement with different heights (Supplementary Fig. 12a). CFD analyses have shown that, after a certain threshold of confinement, the propulsion force generated by a microroller dropped nearly to zero around the confinement entrance, the relative *F*_*P*_ decrease reached to 100% for *h*_*c*_′ = 1.05, indicating the rollers would come to a standstill before entering to the confined region (Supplementary Fig. 12b). Once the relative *F*_*P*_ decrease is 100%, the microrollers cannot translate further at the entrance of the confinement despite their constant rotation (Fig. 2e), explaining the reason why the microroller was not able to enter the confinement.

### Hydrodynamic interactions of the microrollers with circular confinements

**Fig. 5:**
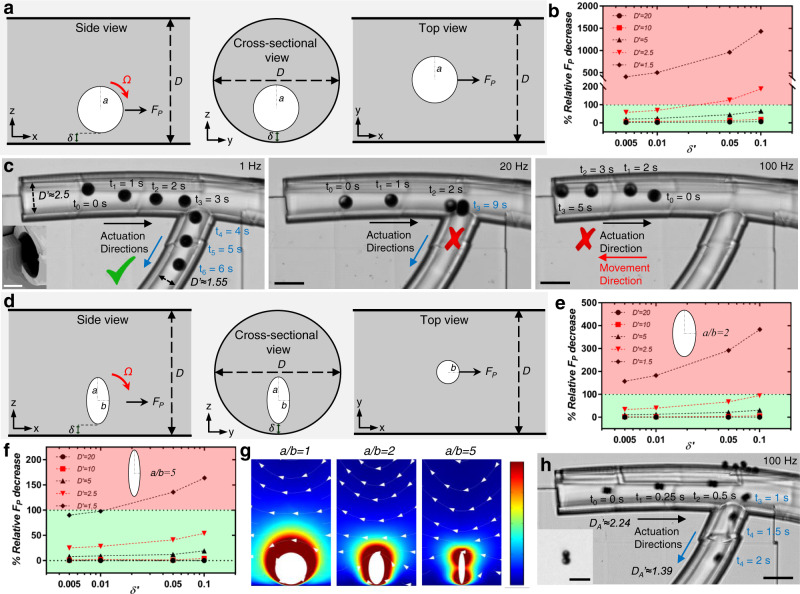
Locomotion of spherical and slender microrollers in confined circular microchannels. **a** A rotating microroller simulated in a cylindrical microchannel, shown from different perspectives. **b** Universal propulsion force decrease depending on *δ’* at different channel diameters (*D*). The force decrease exceeded 100% (red area), meaning the propulsion force reversed at the confined cases at higher *δ*^*’*^. This indicated that the microrollers could reverse their intended translational direction at high frequencies in confined channels. **c** Locomotion in 3D microprinted circular microchannels. The inset in the first image shows the tilted scanning electron microscopy image of the inlet of the circular confinement (Scale bar: 10 μm). The microroller was able to locomote at *f* = 1 Hz in the microchannels with two different circular confinements (*D’* = *D/2a* ≈ 2.5 and 1.55*)*. However, when the frequency was increased to *f* = 20 Hz, while the microroller could still move in the bigger microchannel, it could not enter the smaller one. When the frequency was further increased to *f* = 100 Hz, the locomotion direction of the microrollers was reversed, as predicted in the CFD analyses. The scale bars are 25 μm. **d** Anisotropic-shaped slender microrollers with different anisotropy rates (a/*b* = 2 and 5) were simulated in the same configurations as the spherical case. **e**, **f** Universal propulsion force decrease graphs depending on *δ’* at different channel diameters for *a/b* = 2 and 5. There was a distinct shift from the red area for anisotropic shape compared to the spherical one, indicating a better performance with increasing anisotropy. **g** The flow-fields generated around rotating objects with different anisotropy. The spherical microroller produces more rotational flows than the anisotropic ones, which leads to more interaction with surrounding boundaries. **h** Locomotion of a slender microroller in the same microprinted circular microchannels. The anisotropic microroller was composed of two beads that had a similar size scale to the spherical microroller (inset optical microscope image with the scale bar of 20 μm). The performance of the anisotropic microroller was dramatically better than the single microroller; it was able to locomote at *f* = 100 Hz, where the spherical microroller was locomoting in reverse direction. The scale bar is 25 μm.

Next, we performed additional CFD analyses and experiments to determine how microrollers were affected by circular confinements, which impose confinement in all directions rather than just the vertical (*z-*) axis. For CFD analyses, the microrollers were placed in a circular tube that was sufficiently large from the *x-*axis (40*a*) (Fig. 5a). We give rotational motion to the fixed spheres (i.e., only rotation in the previous section) in different tube diameters and quantified the *F*_*P*_ decrease relative to the planar semi-infinite case (40*a* × 40*a* × 40*a*). The analyses revealed that the confinement effect increased dramatically in circular tubes due to confinement from all directions, especially at smaller diameters (Fig. 5b). While the microroller did not experience any confinement effect in a sufficiently large circular tube compared to the semi-infinite planar case (Fig. 5b, *D’* = *D/2a* = 20), the relative propulsion force, *F*_*P*_ decrease exceeded 100% in smaller tubings at the high frequency (i.e., higher $\delta ^{\prime}$) region. The direction change in *F*_*P*_ (red shaded area in Fig. 5b) indicates that the microroller locomotion could be reversed in small circular tubings at high rotation frequencies.

To test whether the confinement effect was more severe in circular channels as predicted in CFD analyses, we performed experiments in circular tubings printed with two-photon lithography (Fig. 5c and Supplementary Fig. 13, inset). The inner diameter of the tubings was *D’* = 2.50 and 1.55, respectively (Fig. 5c). First, we actuated the microroller at *f* = 1 Hz, and the microroller was able to locomote in both tubings with ease (Fig. 5c, first caption, and Supplementary Movie 3). However, when the actuation frequency was increased to *f* = 20 Hz, the microroller could locomote in the bigger tubing but could not enter the smaller tubing (Fig. 5c, second caption, Supplementary Movie 3). As predicted in CFD analyses, the microroller could not locomote to the intended actuation direction at 100 Hz but reversed its locomotion direction (Fig. 5c, third caption). The microroller oscillated back and forth at *f* = 100 Hz (Supplementary Movie 3), whereby the net displacement was in the reverse direction of the actuation direction. The oscillatory motion was due to the fact that the extents of the confinement effects were different at the center and the lateral side of the cylindrical channels, which caused reverse and forward locomotions in the center and the lateral side of the channel, respectively. We applied the rotating magnetic field in the clockwise-horizontal direction in Fig. 5c, so that the microroller was normally actuated from left to right in the field of view. However, since the cylindrical tube did not have a full straight design in the horizontal direction (Fig. 5c), it had a slightly bent design, and the microroller naturally tended to go to the lateral sides of the tubings when actuated. The microroller experienced different confinement effects at different positions of the tubing, causing forward and reversed locomotion; therefore, it locomoted forth and back. To elucidate the confinement effect in different positions of the tubing, we performed additional CFD analyses (Supplementary Fig. 14a–c). The analyses have revealed that the confinement effect was more severe in the center of the channel, while it was significantly less severe on the lateral sides of the channel, showing the lateral sides of the channel are more advantageous for more efficient locomotion and microroller could perform forward locomotion at these sites (Supplementary Fig. 14d). Additionally, we performed a set of careful experiments to carefully investigate the applicability of the results from the CFD analyses. We microprinted fully straight cylindrical tubing in a similar inner diameter in Fig. 5c (*D’* = 2.5*)*. When actuated in the center of the channel at 100 Hz in the horizontal direction, the microroller reversed its locomotion direction without any oscillation, while the ones in the unconfined region in the same field of view locomoted to the intended actuation direction (Supplementary Fig. 15a, Supplementary Movie 3). On the other hand, when actuated at the lateral side of the channel, the microroller was able to move to the intended actuation direction with a very low translational speed, 5.55% of the unconfined case at 100 Hz. Overall, we conclude that the oscillatory motion was due to microrollers having different locomotion abilities in different positions of the tubing, namely, their locomotion direction changed based on their positions (Supplementary Fig. 15b, Supplementary Movie 3). We also demonstrated that the observed effects were not due to the step-out of the microrollers under the severe circular confinement (*D’* = 2.5*)*. We have observed that the Janus caps of the microrollers at the cylindrical tubing and unconfined region (*h*_*c*_′ = 8.88) were synchronously rotating with the applied magnetic rotational field (Supplementary Fig. 16, Supplementary Movie 3)

From the CFD analyses in the previous section, we found out that the out-of-plane rotational microflows were the main contributing factor to the slowdown of the microrollers in confined spaces. Therefore, a microroller creating a smaller rotational flow field would be more efficient in confined spaces since smaller flow-fields would interact less with the surrounding boundaries. To test the validity of this hypothesis, we performed CFD analyses with rotating ellipsoid structures, generating smaller rotational flow-fields, in the same tubing configuration (Fig. 5d). The analyses revealed that the performance of the microrollers in circular confinements increased with increasing anisotropy (Fig. 5e, f), due to the smaller rotational flow generated (Fig. 5g). We also performed additional CFD analyses with the same ellipsoids in a semi-infinite planar space, and the same trend was also valid compared to the spherical structure (Supplementary Fig. 17 and Fig. 5g). Of note, even though the microrollers with increasing anisotropy were shown to have better performance in CFD analyses, increasing the anisotropy after a certain degree causes an increase in relative repulsion force from the nearby wall^31^, which also causes increased lubrication distance. With increased $\delta ^{\prime}$, the locomotion efficiency of the microrollers would also be lower (Fig. 4f), which causes impeded locomotion, as experimentally shown previously in magnetic microrods having nanometer-ranged diameter^32^. Therefore, increasing the anisotropy after a certain threshold would cause impeded locomotion in experiments.

To validate our CFD analyses with the experiments, we used an anisotropic/slender microroller with the same length as the spherical microroller, creating smaller flow-fields^14^. Anisotropic microroller was composed of two self-assembled Janus particles (Fig. 5h, inset), each having 1000 nm Ni and 50 nm Au on a 5 μm silica particle with an average length of 12.02 μm (Supplementary Fig. 18a). The translational speed of the anisotropic microrollers was similar to the spherical microrollers (Supplementary Figs. 18b and 3) under no confinement. Therefore, the spherical and anisotropic microrollers were comparable; the anisotropic microroller had a slightly higher translational speed due to its higher long axis/length (12.02 μm) under no confinement (Supplementary Figs. 18 and 3). The anisotropic microroller was able to move freely in both large and small circular microchannels at *f* = 100 Hz (Supplementary Movie 3) without a step-out effect (Supplementary Fig. 19), where the spherical microroller could not even locomote (Fig. 5c and Supplementary Movie 3). The experimental results verified our hypothesis along with the CFD analyses; namely, a microroller creating smaller out-of-plane rotational flows, thus less hydrodynamic interactions was more efficient in confined spaces.

## Discussion

In this study, we have demonstrated that the out-of-plane rotational microflows slowdown the motion of the surface microrollers in confined spaces at high rotation frequencies due to the increased hydrodynamic interactions with the surrounding boundaries. Spherical microrollers, creating relatively larger rotational flow-fields, experienced the confinement effect to a higher extent. On the other hand, the slender/anisotropic microrollers, creating smaller rotational flow-fields, demonstrated greater performance in confined spaces thanks to the favorable hydrodynamic interactions. At the same time, the translational fluid flows created by the microrollers had much less effect on the decreased translational velocity of the microrollers. The findings presented here provide an essential understanding of the hydrodynamic interactions of microrobots with their microenvironments, which are crucial not only for the microrollers but also for other microrobots as well. For instance, one can design effective propulsion schemes that do not include rotational or orthogonal flows, interacting less with the surrounding environments.

One of the most important application areas of microrobots is a medicine; they can be used in applications such as targeted drug/gene delivery^33,34^ thanks to their active targeting capability. Many biological media, including blood vessels, and tissues, exhibit microtopographies and confinements that are very different from microfluidic chips used in all in vitro experiments. Only the microrobots with optimal flow generation would navigate in such complex body environments. The results presented here show that microrobot locomotion can be severely affected under microenvironments with extreme confinement, which could be very well encountered in biological environments such as venules, capillaries, and porous matrices. Thus, understanding the hydrodynamic barriers and developing alternative strategies are important steps to achieve efficient locomotion of the microrobots in the body toward future high-impact medical applications.

Designing microrobots with programmed flow generation is a promising research direction; such microrobots can be fabricated by utilizing advanced 3D fabrication techniques^9,35–38^ and actuated by external stimuli such as acoustic fields^9,37,39–41^ for efficient locomotion in narrow spaces. In addition, rather than designing individual microrobots, the performance of microroller swarms in confined environments is an interesting aspect that is worth investigating. The rotational flows generated by individual microrollers could actually be useful to locomote in small spaces; the generated flows could push the consecutive microrollers and enable efficient locomotion. Microroller locomotion in confined spaces inside non-Newtonian fluids^42–47^ is another interesting area that is currently unexplored. Other than that, understanding the hydrodynamic interactions of surface microrollers with their surroundings could also help to elucidate the behavior of the colloidal systems found under the same conditions (e.g., rotating colloids in microchannels or porous media) with their plethora of applications in soft matter, chemical engineering, and nanotechnology^48–50^. Overall, the physical principles that are unraveled here will be essential to modify the conventional propulsion schemes toward real-world applications of microrobots.

## Methods

### Fabrication of surface microrollers and their magnetic actuation

Magnetic spherical Janus microrollers were fabricated by sequentially sputtering 1000 nm Ni and 50 nm Au nanofilms on predried silica (SiO_2_) particles with 10 μm diameters (microParticles GmbH) using a sputter coating system (Leica EM ACE600, Leica Microsystems). The silica particles were prepared in a 1% w/v in deionized (DI) water, and 250 μL of particle suspension was drop cast onto a plasma-treated 2 × 2 cm^2^ glass slide. After drying in the ambient environment, the abovementioned thickness of Ni and Au were deposited on the particles using the sputter system. The magnetization direction of the microrollers was programmed to out-of-plane direction by using a 1.8 T uniform magnetic field in a vibrating sample magnetometer (MicroSense, Lowell, MA). After, glass slides were placed in a 30 mm diameter glass petri dish and filled with 2 mL ethanol. The glass petri dish was placed in a sonicator bath, the sonication of the petri dish allowed particles to move to the ethanol phase in a few seconds. The particles in ethanol were then collected in a 2 mL centrifuge tube, the media was replaced with a phosphate-buffered saline (PBS) 1×. The abovementioned steps were also used to fabricate doublet microrollers using SiO_2_ particles with the 5 μm diameter (microParticles GmbH). Finally, the doublet microrollers were formed in PBS 1× by mixing the particles at relatively low concentrations^14^. All experiments were performed in PBS 1×. The microrollers were actuated using a custom-made five-coiled electromagnetic coil system placed on a microscope (Zeiss Axio Observer A1, Carl Zeiss). The microrollers were actuated using uniform rotating magnetic fields with an amplitude of 10 mT that enables surface rolling and steering control. We characterized the magnetic properties of the microrollers with a vibrating sample magnetometer (Supplementary Fig. 20). The analyses revealed that the coercivity of the particles was around 15 mT. For the basic vertical confinement analysis, at least 16 microrollers were recorded and tracked. The videos were recorded using a high-speed camera (M310; Phantom, Inc.). The images were then analyzed using a Trackpy Python package to determine the velocity of the microrollers. At least 3 and 20 microrollers were analyzed to assess the synchronous rotation of the microrollers with the applied field for planar and circular confinements, respectively.

### Fabrication of confined microchannels

Planar-confined microchannels were fabricated using spacer particles with different diameters (16.94, 25, 50, and 100 μm diameters, microParticles GmbH, Corpuscular Inc.). 1 mL of particle solutions were dried using a centrifuge tube heater at 65 °C. Then the dried spacer particles were first embedded into 1 mL of UV glue, and then a drop of the spacer particle-containing UV glue was applied to the precleaned glass surface from different points. Another precleaned glass surface was placed on it, and the sandwich structure (Fig. 2a) was formed using clamps for UV curing for 15 min. Clamps were removed after curing the glue, and the microrollers were injected from the available sides of the channels using a micropipette. After the injection, the microchannels were fully sealed with additional glue to ensure that the system was fully closed.

Step-like and irregular vertical confinement channels were fabricated from IP-S photoresist using a commercially available two-photon lithography system using 63× objective (Photonic Professional, Nanoscribe GmbH). The experiments were performed in closed microchannels composed of two glass slides and the laser-cut, 50 μm thick double-sided adhesive. The top glass slide encloses the fluidic connections, the thickness of the double-sided adhesive defines the channel height, and the other glass side makes the basement. The structures were microprinted on the top channels to create local confinements. The height and the 3D profile of the fabricated microstructures and channels were measured with an optical profilometer to get precise information about the structures (VK-X250, Keyence).

The circular confinements were also microfabricated using a two-photon lithography system using 63× objective (Photonic Professional, Nanoscribe GmbH). The 3D computer-aided design files were generated in SolidWorks (Dassault Systèmes). All structures microprinted with a two-photon lithography system in the study were developed with a standard developer, propylene glycol methyl ether acetate (Sigma–Aldrich), for at least 1 h. Scanning electron microscopy imaging of the 3D-printed structures and microrollers was performed via a ZeissUltra 550 Gemini scanning electron microscope (Carl Zeiss Inc.).

### Computational fluid dynamics (CFD) analyses

COMSOL Multiphysics 5.5 Simulation Software (COMSOL, Inc.) was used to simulate microrollers and calculate the forces acting on the microrollers in all conditions, using creeping-low interface physics by solving the Navier-Stokes equations. The simulations were performed in 3D, where the mesh around the microroller was refined until converging to results from Goldman et al.^22^. We defined two different mesh regions, the first one is around the microroller and the second one covered the remaining spaces in the simulation environment. The dimension of the mesh around the microroller was 3*a* × 3*a* × 3*a*, where minimum element size, maximum element size, maximum element growth rate, curvature factor, and the resolution of narrow regions were defined in COMSOL 0.05 μm, 1 μm, 1.5, 0.2, and 1.5, respectively (Supplementary Fig. 21). For the second mesh, the values were 4 μm, 13.4 μm, 1.15, 0.6, and 0.7, respectively. For the vertical confinement analyses, after determining 40*a* × 40*a* × 40*a* corresponds to the semi-infinite case, the channel dimensions with 40*a* × 40*a* × *h*_*c*_, were used where *h*_*c*_ determines the degree of confinement. For circular confinements, the microroller translational movement axis was kept at 40*a*, while the channel diameter specified the other dimensions. For elliptical microrollers, the forces were calculated by taking the average of the forces over one cycle. The bodies were rotated from the center of the objects, while the distance from the center to the nearby wall was kept the same (*δ* *+* *a*) throughout the rotation. The boundaries other than microrollers were defined as no-slip boundaries, and the microroller was actuated in DI water.

### Statistical analysis

All quantitative experimental values were presented as means ± SD of the mean.

## Supplementary information

Supplementary Information

Description of Additional Supplementary Files

Supplementary Movie 1

Supplementary Movie 2

Supplementary Movie 3

## Data Availability

All data are available from the corresponding author upon reasonable request.

## Code availability

All relevant codes are available upon request from the corresponding author.
