# Supplementary material for: Reduced rotational flows enable the translation of surface-rolling microrobots in confined spaces
Source: Nat Commun. 2022 Oct 21;13:6289. doi: 10.1038/s41467-022-34023-z (PMC9586970; doi:10.1038/s41467-022-34023-z)
Supplement: Supplementary file 1 — Supplementary Information [file 41467_2022_34023_MOESM1_ESM.pdf]

SUPPLEMENTARY INFORMATION

for

Reduced rotational flows enable the translation of surface-rolling microrobots in confined spaces

Ugur Bozuyuk[1,2], Amirreza Aghakhani[1], Yunus Alapan[1], Muhammad Yunusa[1], Paul Wrede[1,2], Metin Sitti[1,2,3]*

[1] Physical Intelligence Department, Max Planck Institute for Intelligent Systems, 70569 Stuttgart, Germany

[2] Institute for Biomedical Engineering, ETH Zurich, Zurich 8092, Switzerland

[3] School of Medicine and School of Engineering, Koç University, Istanbul 34450, Turkey

* Correspondance to: sitti@is.mpg.de

Supplementary Figures

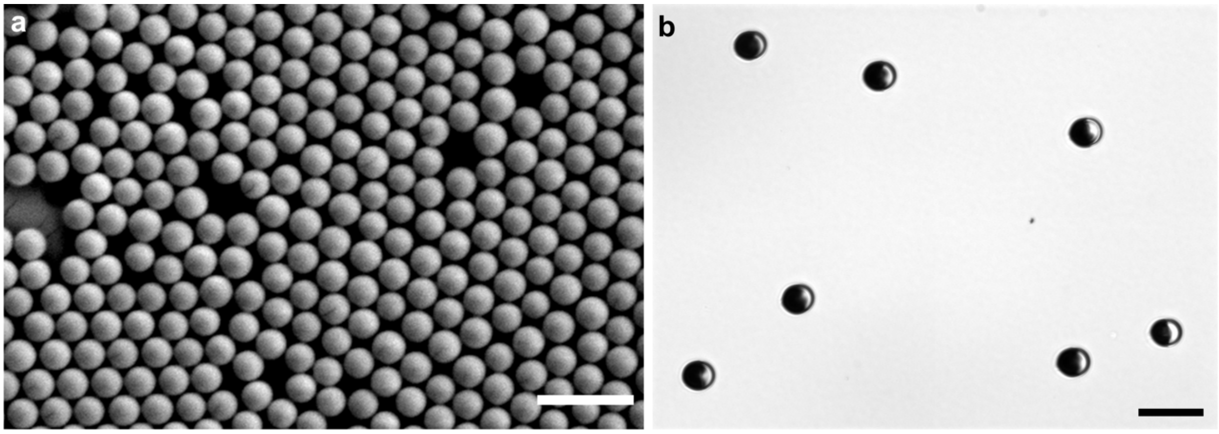

Supplementary Figure 1. The spherical Janus microrollers used in the experiments. a) A scanning electron microscopy image of the microrollers. The average diameter from the top view is 10.26 ± 0.55 µm. The scale bar is 30 µm. **b)** Light microscopy image of the Janus microrollers. The average diameter from the side is 11.37 ± 0.36 µm. The scale bar is 25 µm. Overall, the average size of the particles was assumed to be 10.8 µm.

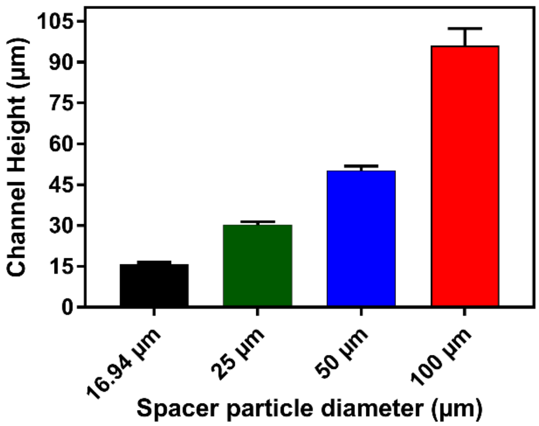

Supplementary Figure 2. Characterization of the fabricated confined microchannels. 3D laser confocal microscopy analysis for the microchannels. The average diameters of the commercially available particles define the channel height. There was a slight difference between particle diameters with the measured channel heights since the spacer particles were not completely monodisperse. At least 5 different channels were analyzed for each group. The error bars show the standard deviation of the mean.

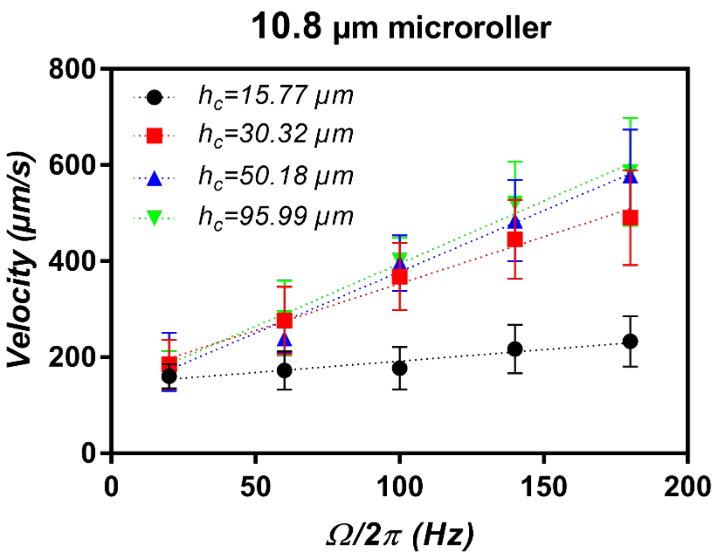

Supplementary Figure 3. Frequency-dependent average translational velocity of microrollers in different confinements at 10 mT field amplitude. The microrollers were not stepped-out in any confinement, demonstrating the decreased velocity trend in the experiments was not due to step-out. The error bars show the standard deviation of the mean.

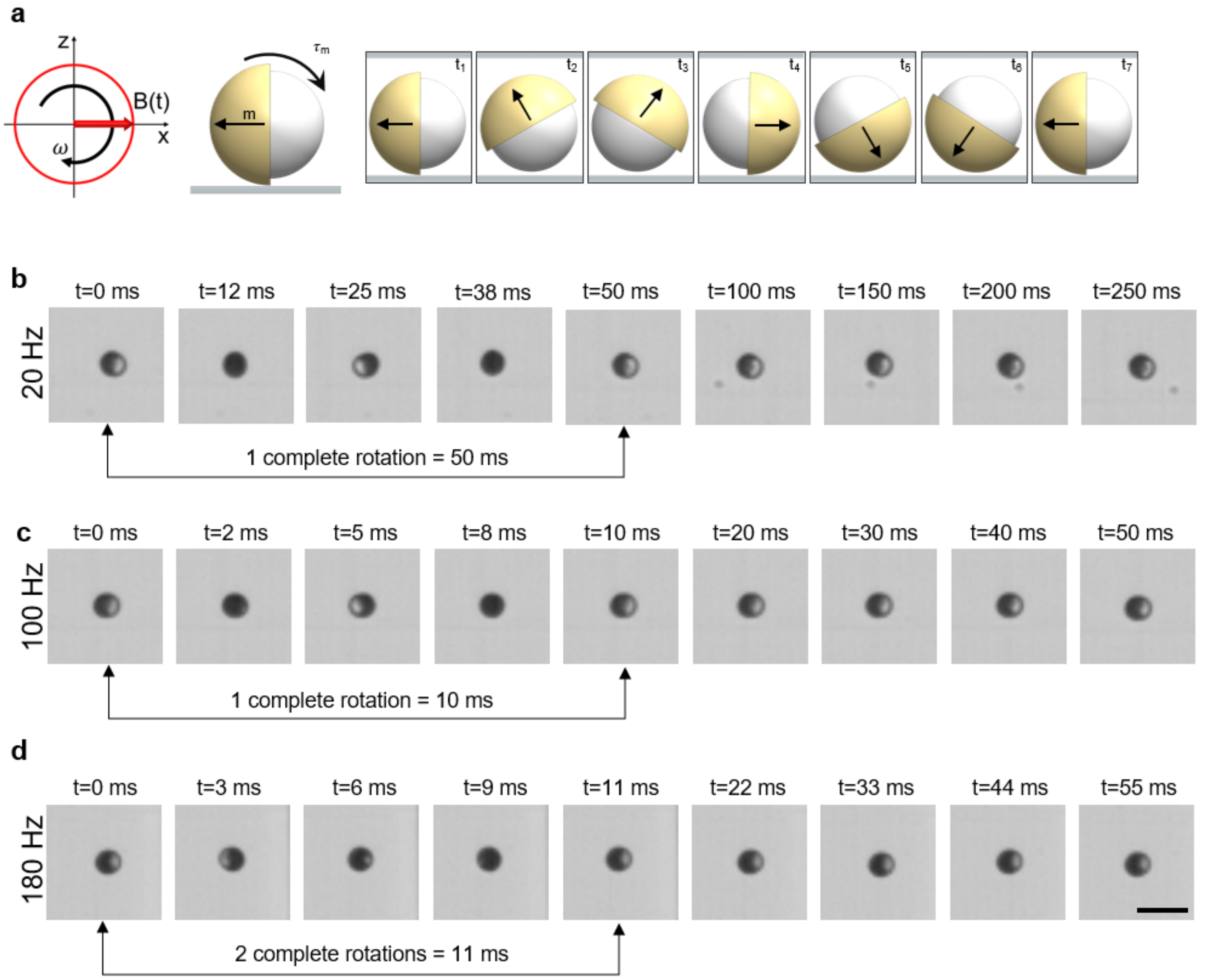

Supplementary Figure 4. Representative 1000 frame-per-second time-lapse images of microrollers under h_c'= 1.22 at 20, 100, and 180 Hz. a) A schematic demonstrating the motion of a microroller from side view. The microrollers are rotated with a rotating magnetic field, where the magnetic cap with magnetic moment m follows the rotating field if the microroller works synchronous rotation regime. It completes a full rotation in a known time, depending on the frequency input. **b)** A microroller locomoting under severe confinement at 20 Hz. Per 50 frames or 50 ms, the microroller finishes one complete rotation, thus, its appearance under a microscope should be similar if it is working in a synchronous rotation regime. The Janus cap positions of the microroller were the same per 50 ms, demonstrating the microroller was not stepped-out. **c)** A microroller at locomoting with 100 Hz under the same confinement. Per 10 ms, the microrollers completes one complete rotation, and cap positions were the same. **d)** At 180 Hz, per ~11 ms, the microroller completes two complete rotations. The scale bar is 20 μm.

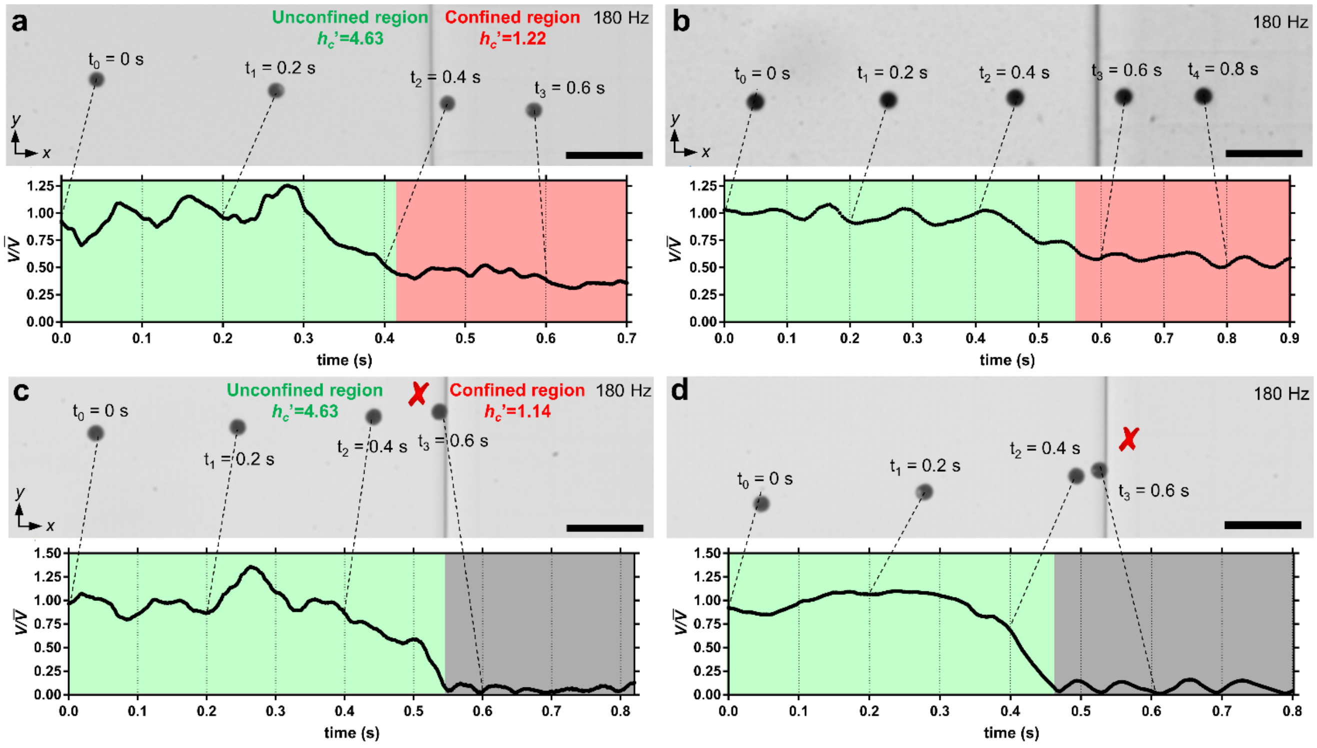

Supplementary Figure 5. The additional set of experiments for step-like local confinements. a-b) The velocity of the microroller dramatically reduced upon entry into the confined region ($h_c{}'= h_c/2a = 1.22$). **c-d)** When the confinement was more severe ($h_c{}'=1.14$), the microroller's velocity dropped to zero and could not even enter the confined region. The instantaneous velocity of the microrollers was normalized to the average velocity of the microrollers in less confined region $h_c{}'$= 4.63. All scale bars are 50 µm.

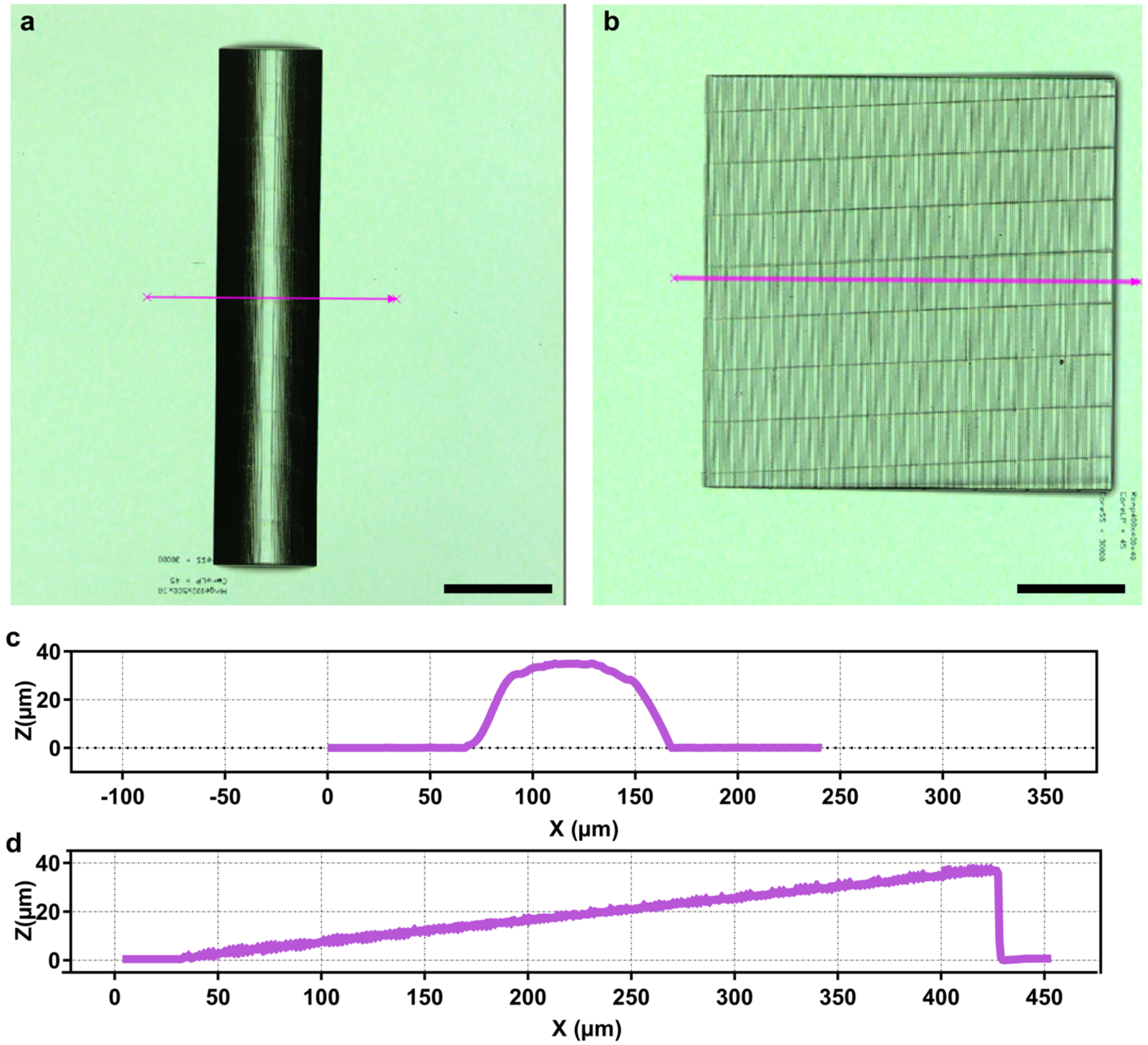

Supplementary Figure 6. 3D laser confocal microscopy images of irregular confinements. a,b) Image of the 3D microprinted half sphere- and ramp-like structures used in the experiments. The colored lines show the position of the profiling analyses. The scale bars are 100 µm. **c,d)** Cross-sectional profiling analyses for the structures used in the experiments. The total channel height was 50 µm, and the structures were on top of the channel, so the difference between total channel height and structure heights determined the degree of confinement.

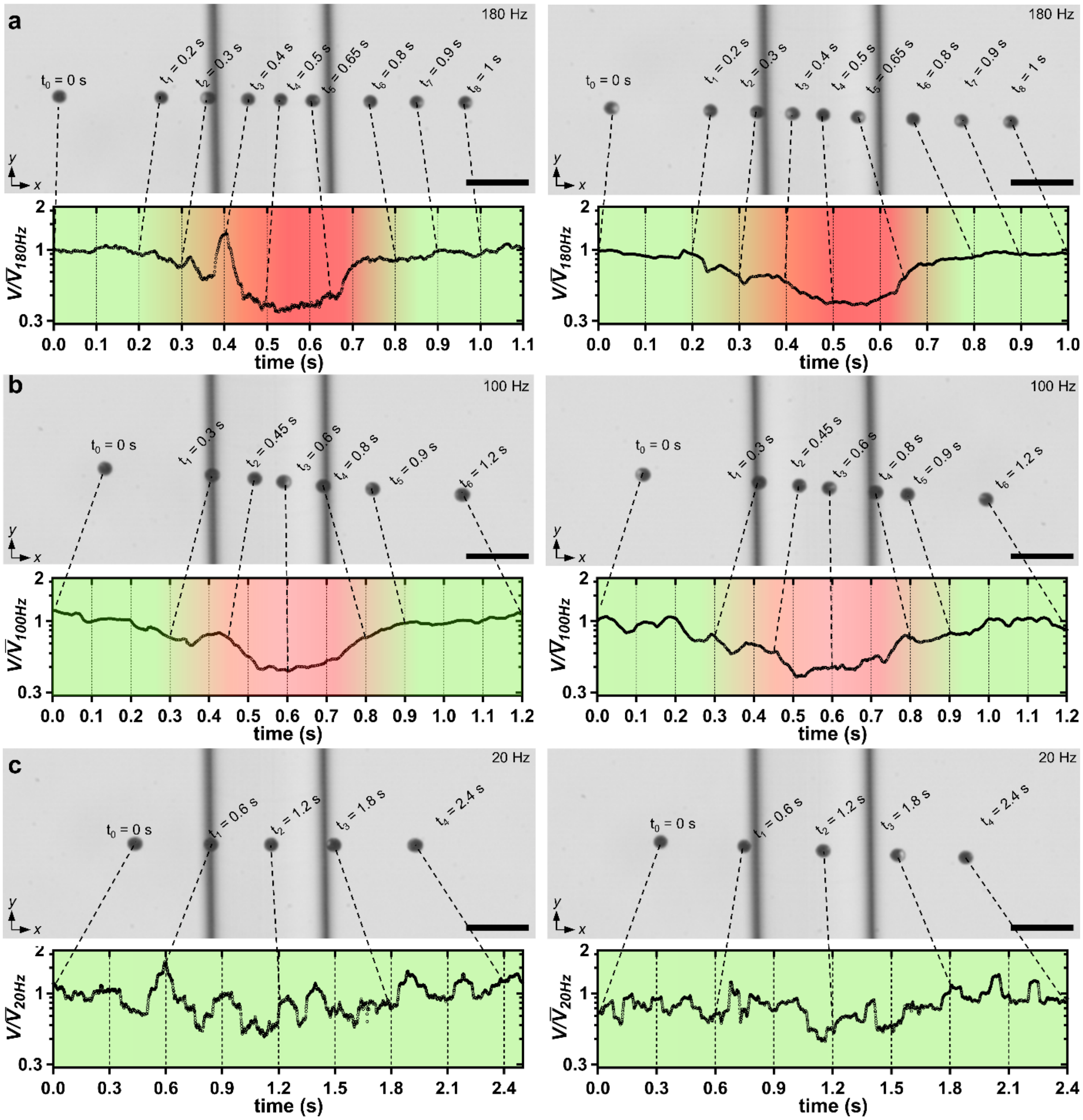

Supplementary Figure 7. 2nd and 3rd set of experiments for microroller locomotion experiments under half sphere-like confinements. a-b-c) The time-lapse images and time versus transitional velocity graphs of microrollers actuated at different rotational frequencies, 180, 100, and 20 Hz, under the half sphere-like confinement. A similar behavior observed in Fig. 3. The instantaneous velocities were normalized to the average velocity for all graphs at the specific condition. All scale bars are 50 µm.

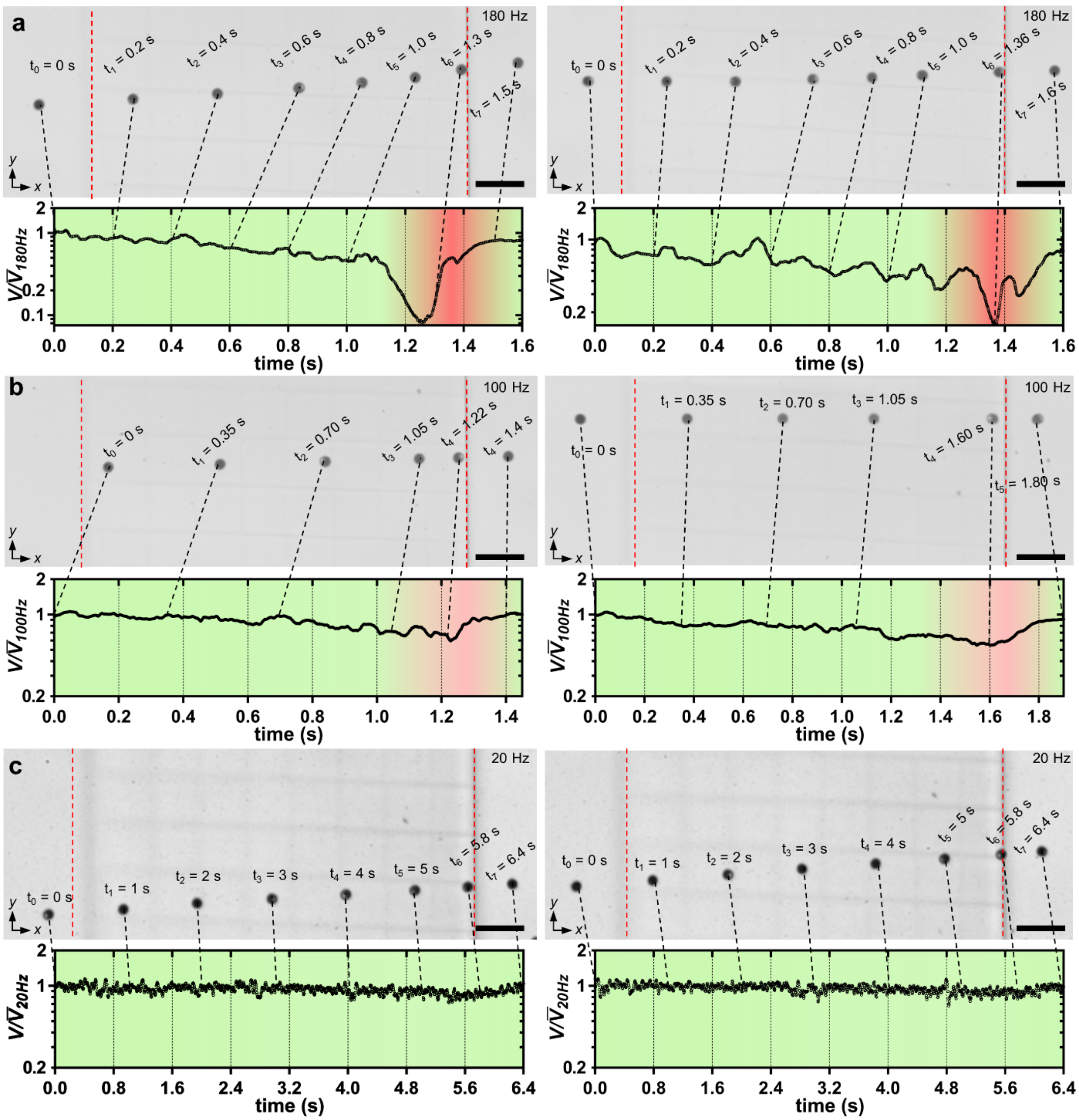

Supplementary Figure 8. 2nd and 3rd set of experiments for microroller locomotion experiments under ramp confinements. a-b-c) The time-lapse images and time versus transitional velocity graphs of microrollers actuated at different rotational frequencies, 180, 100, and 20 Hz, under the ramp confinement. A similar behavior observed in Fig. 3. The instantaneous velocities were normalized to the average velocity for all graphs at the specific condition. All scale bars are 50 μm.

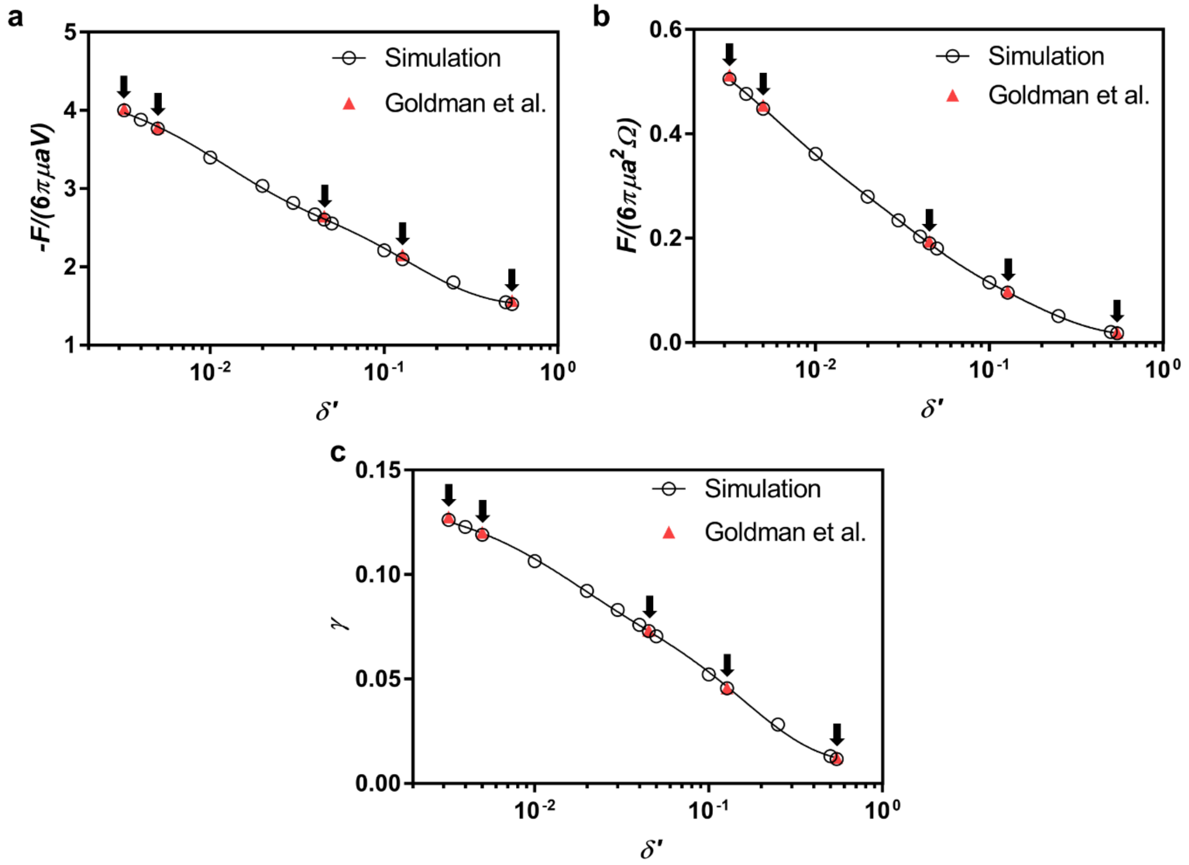

Supplementary Figure 9. Validation of the CFD simulations with the results reported by Goldman et al.[22] a) Normalized force on a translating sphere, **b)** Normalized force on a rotating sphere as a function of *δ'***. c)** Slipping coefficients as a function of *δ'***.** The results from Goldman et al. were labeled with black arrows in all graphs.

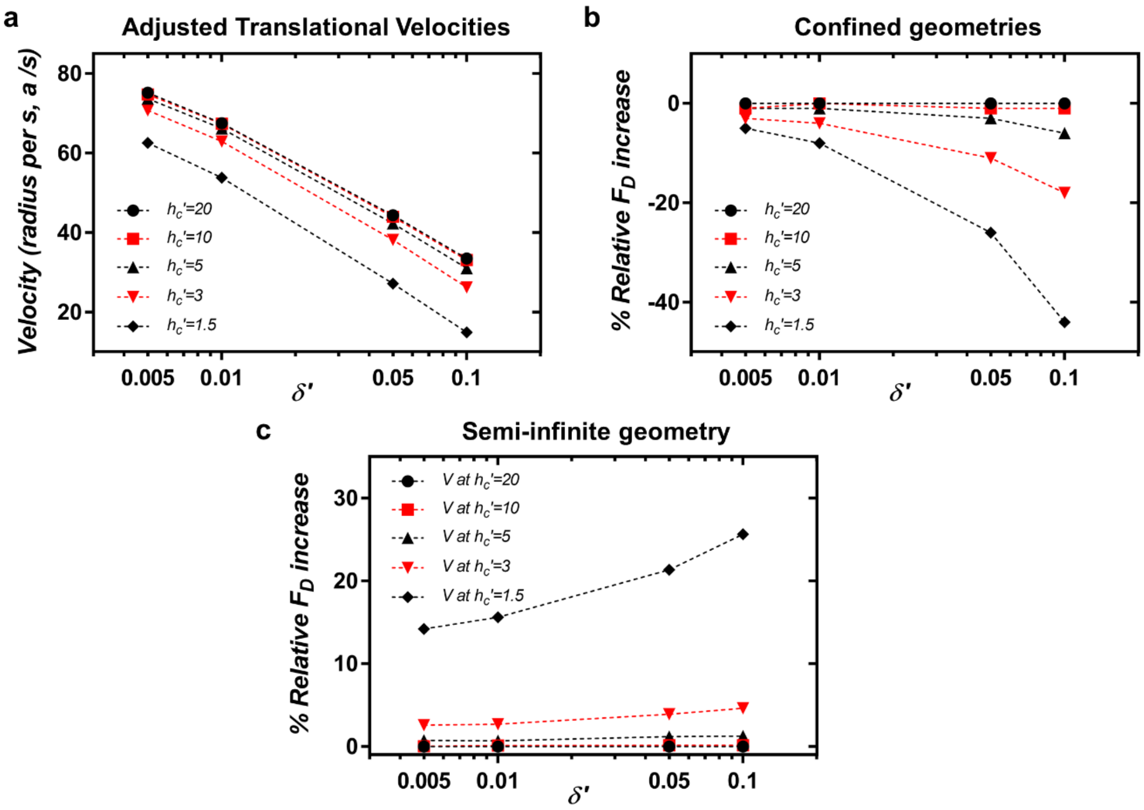

Supplementary Figure 10. Simulated translation of a spherical object in confined spaces with adjusted speeds according to the propulsion force (*F_P*) decrease. a) The calculated theoretical speeds in different confined spaces with respect to *δ'*. The speeds are given in a normalized manner, radius length/second. **b)** Universal drag force increase graph to *δ'* under different confinements of a translating sphere with adjusted speeds, given in **(a)**. Instead of increasing F_D, it decreased because of decreased calculating speeds. This shows the main contribution of the decreasing speeds in the experiments was the rotational flows. **c)** The microrollers with adjusted speeds were simulated in the semi-infinite case ($40a \times 40a \times 40a$), and the values were relatively compared to the ones in **(b)**. The difference between the two distinct cases demonstrates the real contribution of the confinement translational drag.

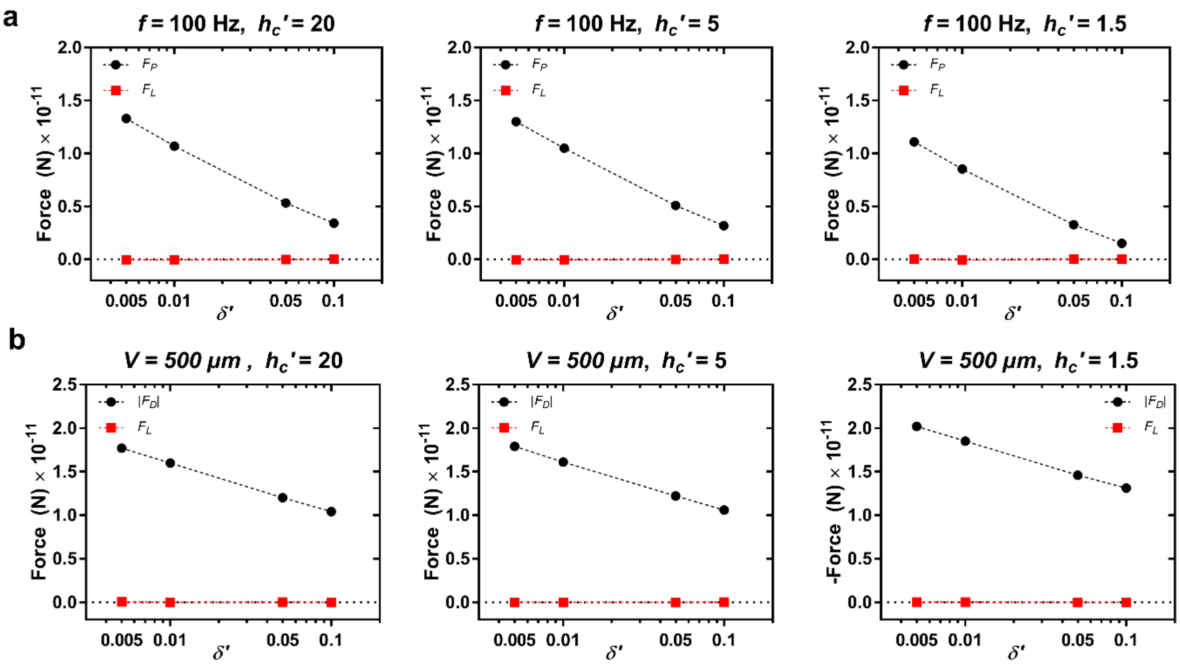

Supplementary Figure 11. The effect of the lift force in CFD simulations. a-b) The z-component of the forces acting on the microrollers were quantified in different lubrication distances at different confinement ratios at f=100 Hz and V=500 μm. In all cases, the relative magnitude of the lift force is negligible compared to the propulsion or drag force.

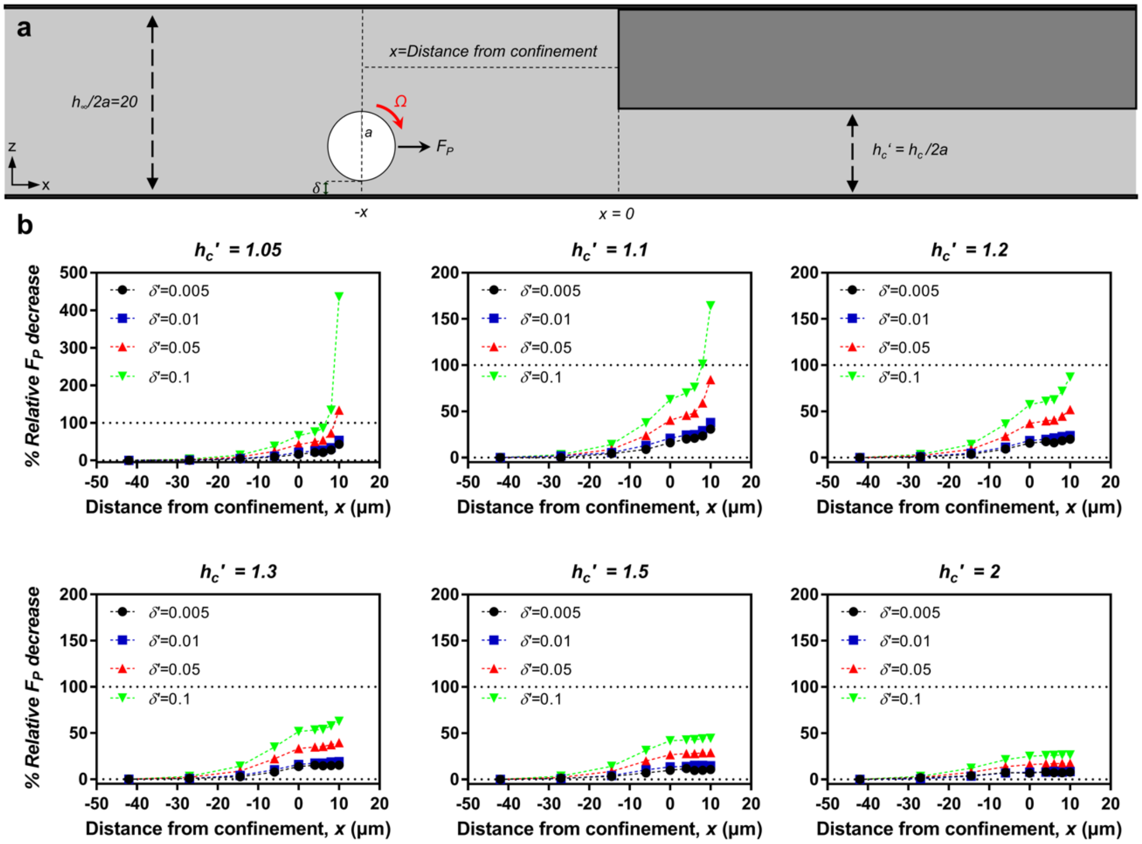

Supplementary Figure 12. The confinement effect on a microroller approaching step-like confinement in CFD simulations. a) A microroller was gradually approached step-likes confinement and the decrease in the propulsion force was quantified. **b)** The analyses for different h_c' revealed that the propulsion force could significantly decrease, and even could result in a nearly 100% decrease just entrance of the step-like confinement, implying its translational velocity could reach zero.

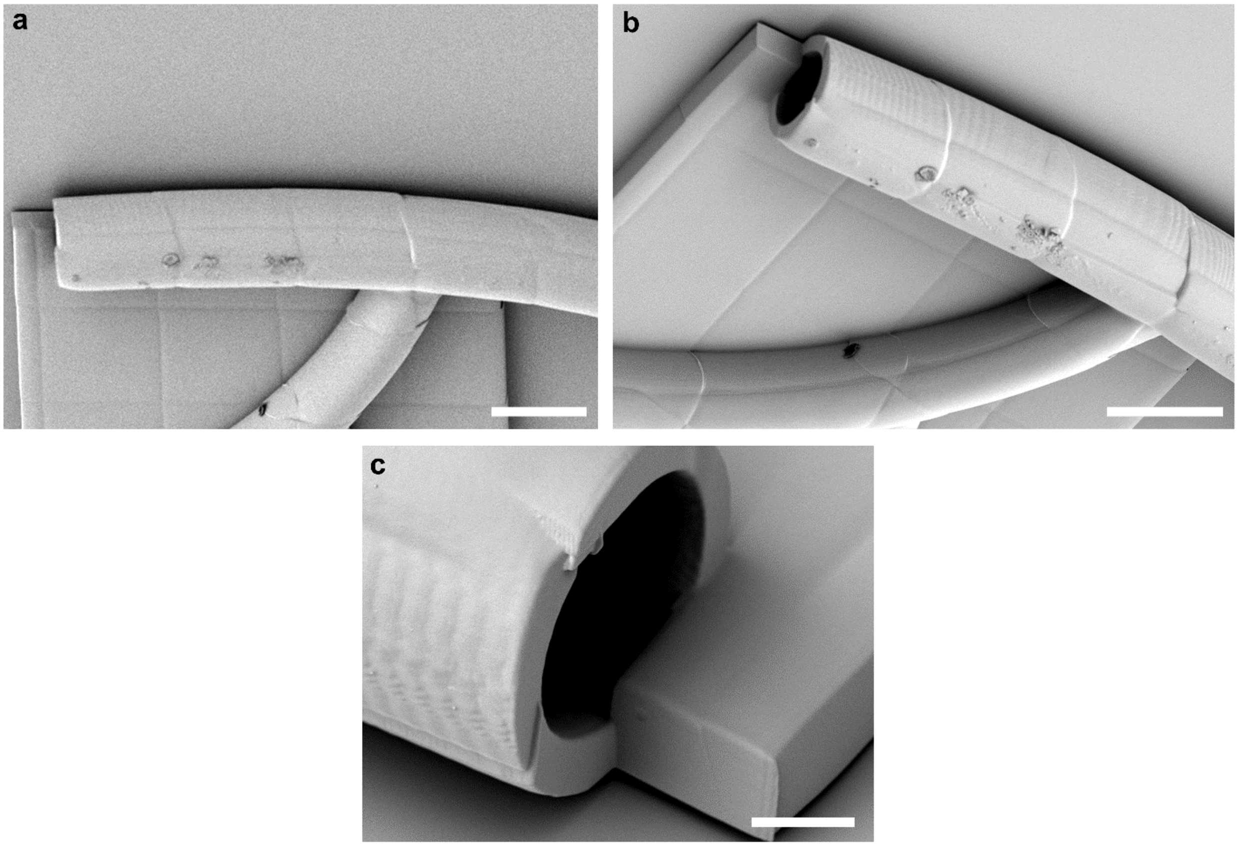

Supplementary Figure 13. Scanning electron microscopy images of the 3D-microprinted circular channels. a,b) The overview of the circular channels from different perspectives. The scales bars are 40 μm. **c)** A close-up image of the circular inlet. The scale bar is 10 μm.

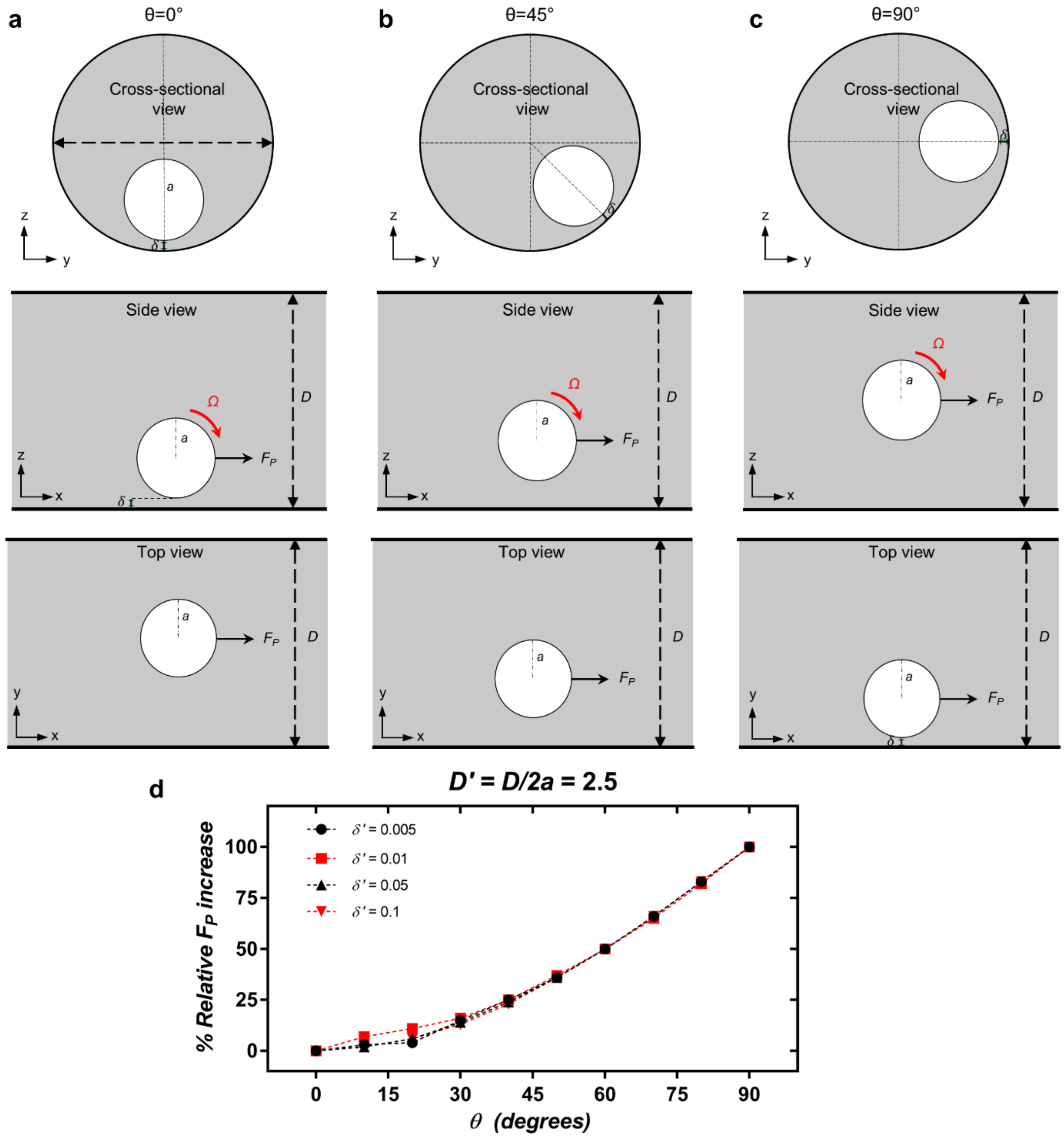

Supplementary Figure 14. Position-dependent propulsion force increase of microrollers in circular confinements. a-c) The position of the microroller was changed from the bottom of the channel **(a,** θ=0**)** to the lateral sides (**b-c,** θ>0) in the simulation environment to assess the effect of the confinement. **d)** The simulations have shown that the microroller's propulsion force increased on the lateral sides, relative to the θ=0 at *D'*=2.5. This, therefore, means that the microroller locomotion is more advantageous at the lateral sides of the circular confinement.

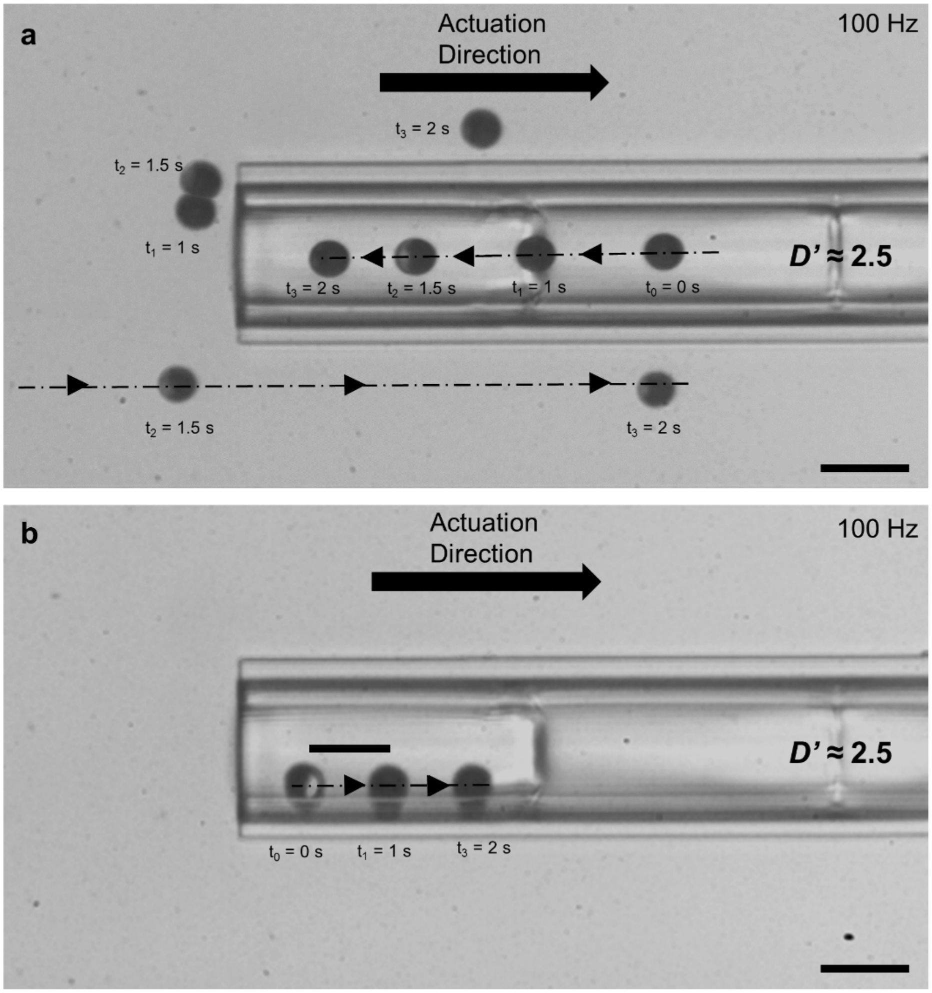

Supplementary Figure 15. Experimental demonstration of position-dependent propulsion force increase of microrollers in circular confinements. a) When actuated at the center of the circular confinement, the microroller locomoted the reverse direction of the actuation direction, while the unconfined microrollers actuated to the intended actuation direction. **b)** The microroller was carefully moved to the lateral side of the channel with 1 Hz and then actuated with 100 Hz. The microroller was able to move to the intended actuation direction with a much slower translational velocity. The experimental results support the simulation results given. The scale bar is 25 μm.

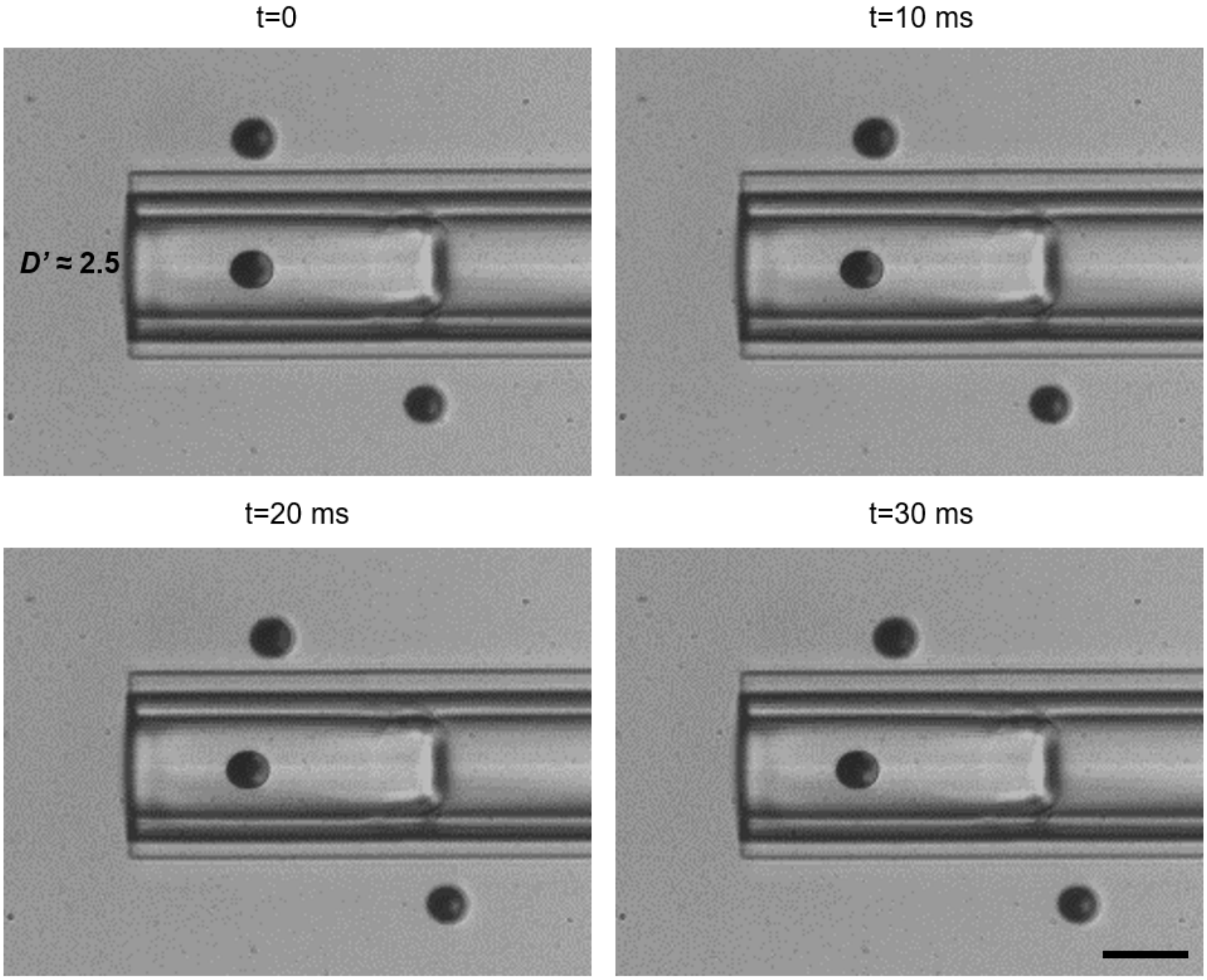

Supplementary Figure 16. 1000 frame-per-second video of microrollers under $D' \approx 2.5$ and unconfined $h_c' = 18.5$ case at 100 Hz. The microroller rotating with 100 Hz should finish one complete rotation per 10 ms if it is in a synchronous rotation regime. In other words, the magnetic Janus cap position has to be the same per 10 ms. In 4 different time points per 10 frames, the microroller inside the circular confinement and the ones outside have shown the same cap positions, demonstrating that the microroller inside the circular confinement was still in the synchronous rotation regime. The scale bar is 25 μm.

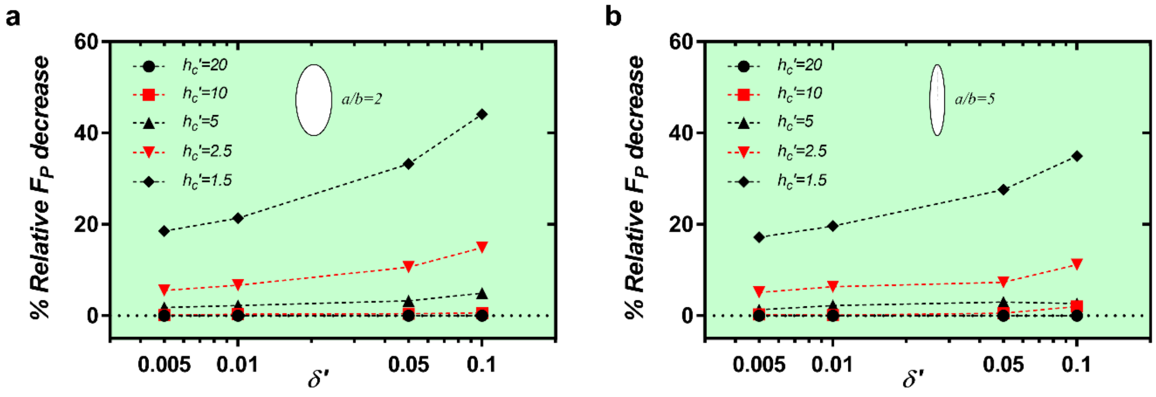

Supplementary Figure 17. Simulated universal propulsion force decrease graphs as a function of δ' at different channel diameters. **a)** a/b = 2 and **b)** 5 in planar confinements. The channel height (h_c) was altered, where the other dimensions were in semi-infinite condition (*40a*). The relative F_P decrease was dramatically less compared to the circular microchannels (**Figure 5e,f**), showing that the confinement from other directions also had a significant impact on the propulsion of a microroller.

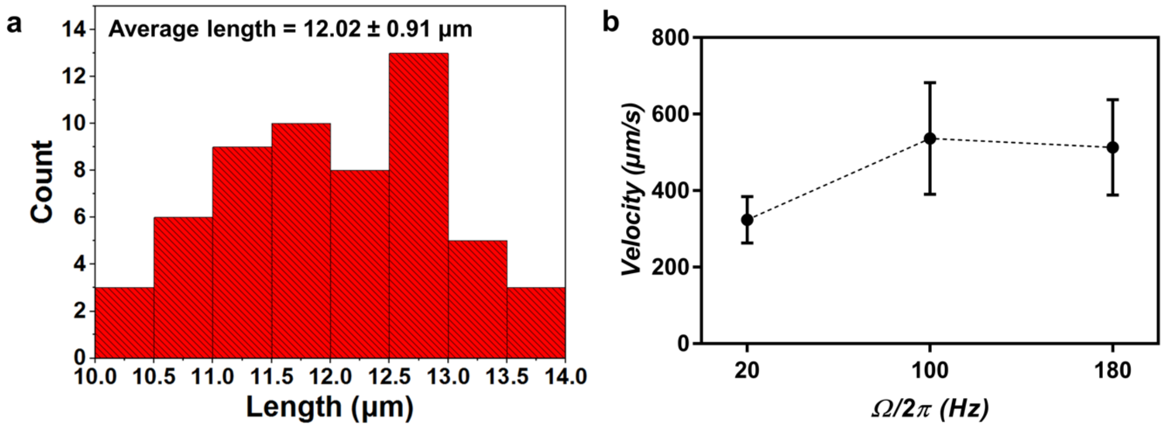

Supplementary Figure 18. Size and speed analysis for the slender doublet microroller. a) The size distribution of the doublet microroller. The average length of the doublet is 12.02 ± 0.91 µm. **b)** Frequency-dependent average translational velocity of microrollers at 100 µm height (semi-infinite) channel. The doublet microroller has stepped out at 180 Hz. Therefore, it had higher average translational velocity than the spherical microrollers at 20 and 100 Hz. The error bars show the standard deviation of the mean.

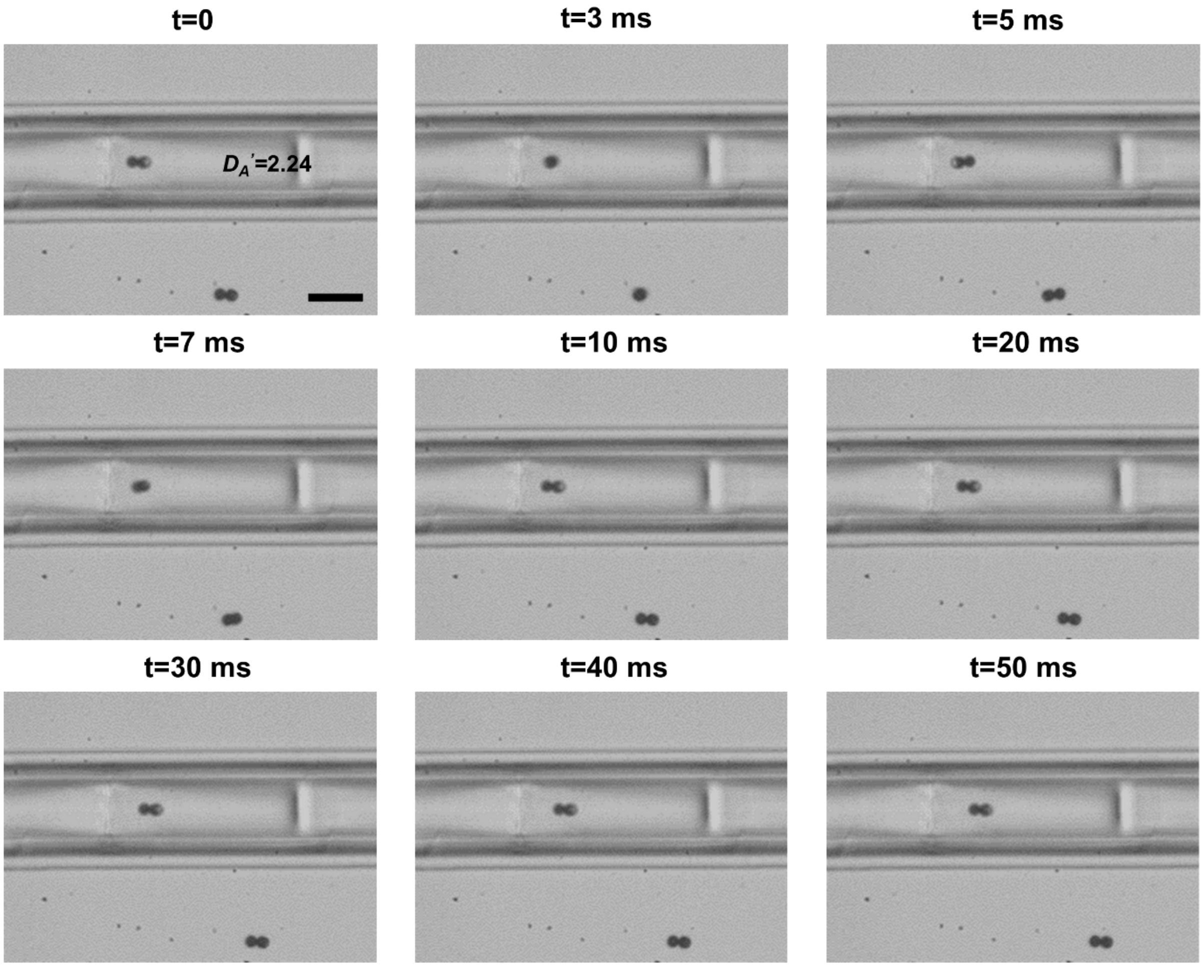

Supplementary Figure 19. 1000 frame-per-second video of anisotropic microrollers under D_A' ≈ 2.24 and unconfined h_c'= 16.55 case at 100 Hz. The doublet microroller rotating with 100 Hz should finish one complete rotation per 10 ms if it is in a synchronous rotation regime. In other words, microrobot body has to be in the same position per 10 ms. In 4 different time points per 10 frames, the microroller inside the circular confinement and the ones outside had the same body position, demonstrating that the microroller inside the circular confinement was still in the synchronous rotation regime. The scale bar is 25 μm.

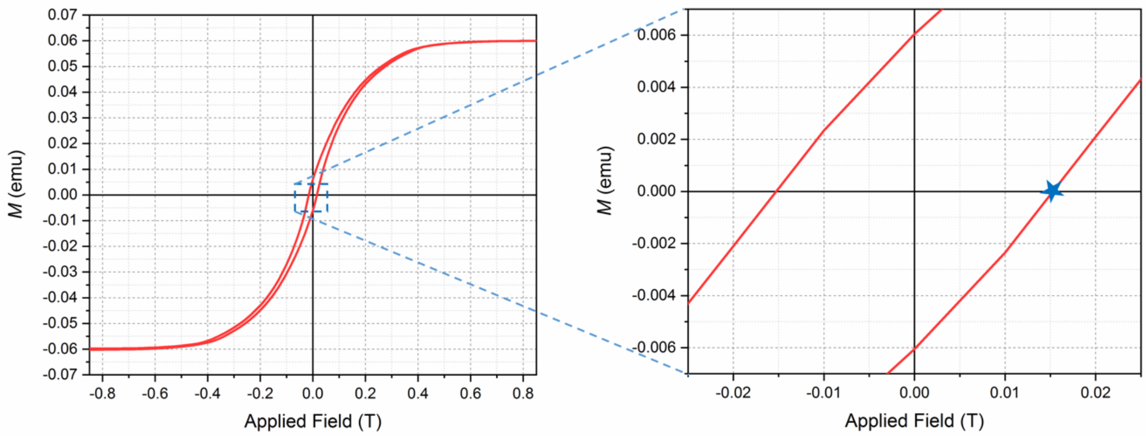

Supplementary Figure 20. Magnetic characterization of Janus microrollers. 2.5 mg of silica microparticles were monolayered on a 2x2 cm^2 glass slide and then coated with 1000 nm Ni and 50 nm Au. The vibrating sample magnetometer (VSM) analysis revealed that the microrollers had ≈15 mT coercivity. This shows that the microrollers behave as hard magnet in the rotating uniform field we worked (10 mT).

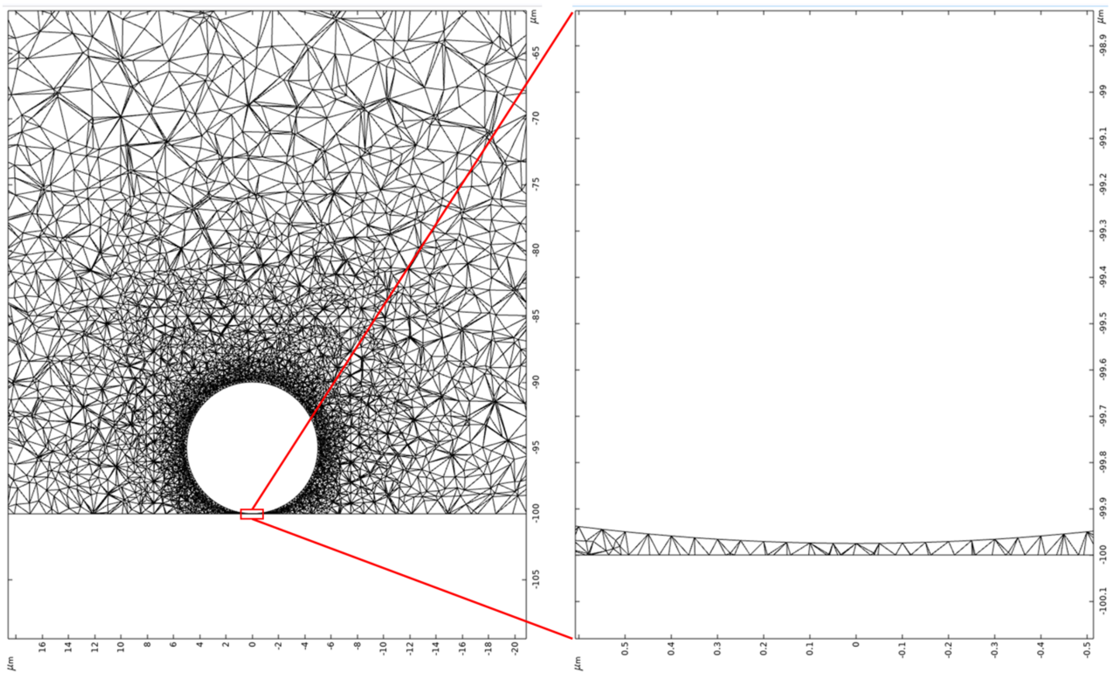

Supplementary Figure 21. A cross sectional image of example mesh configuration for the smallest lubrication distance, $\delta' = 0.005$.
