# Supplementary material for: Reduced rotational flows enable the translation of surface-rolling microrobots in confined spaces
Source: Nat Commun. 2022 Oct 21;13:6289. doi: 10.1038/s41467-022-34023-z (PMC9586970; doi:10.1038/s41467-022-34023-z)
Supplement: Supplementary file 2 — Description of Additional Supplementary Files [file 41467_2022_34023_MOESM2_ESM.pdf]

Description of Additional Supplementary Files

Supplementary Movie 1 Microrollers under step-like vertical confinements
Supplementary Movie 2 Microrollers under irregular vertical confinements
Supplementary Movie 3 Microrollers in circular confinements
